# Copy Number Variation Is a Fundamental Aspect of the Placental Genome

**DOI:** 10.1371/journal.pgen.1004290

**Published:** 2014-05-01

**Authors:** Roberta L. Hannibal, Edward B. Chuong, Juan Carlos Rivera-Mulia, David M. Gilbert, Anton Valouev, Julie C. Baker

**Affiliations:** 1 Department of Genetics, Stanford University School of Medicine, Stanford, California, United States of America; 2 Department of Biological Science, Florida State University, Tallahassee, Tallahassee Florida, United States of America; 3 Division of Bioinformatics, Department of Preventive Medicine, University of Southern California Keck School of Medicine, Los Angeles, California, United States of America; University of Pennsylvania, United States of America

## Abstract

Discovery of lineage-specific somatic copy number variation (CNV) in mammals has led to debate over whether CNVs are mutations that propagate disease or whether they are a normal, and even essential, aspect of cell biology. We show that 1,000N polyploid trophoblast giant cells (TGCs) of the mouse placenta contain 47 regions, totaling 138 Megabases, where genomic copies are underrepresented (UR). UR domains originate from a subset of late-replicating heterochromatic regions containing gene deserts and genes involved in cell adhesion and neurogenesis. While lineage-specific CNVs have been identified in mammalian cells, classically in the immune system where V(D)J recombination occurs, we demonstrate that CNVs form during gestation in the placenta by an underreplication mechanism, not by recombination nor deletion. Our results reveal that large scale CNVs are a normal feature of the mammalian placental genome, which are regulated systematically during embryogenesis and are propagated by a mechanism of underreplication.

## Author Summary

Generally, every mammalian cell has the same complement of each part of its genome. However, copy number variation (CNV) can occur, where, compared to the rest of its genome, a cell has either more or less of a specific genomic region. It is unknown whether CNVs cause disease, or whether they are a normal aspect of cell biology. We investigated CNVs in polyploid trophoblast giant cells (TGCs) of the mouse placenta, which have up to 1,000 copies of the genome in each cell. We found that there are 47 regions with decreased copy number in TGCs, which we call underrepresented (UR) domains. These domains are marked in the TGC progenitor cells and we suggest that they gradually form during gestation due to slow replication versus fast replication of the rest of the genome. While UR domains contain cell adhesion and neuronal genes, they also contain significantly fewer genes than other genomic regions. Our results demonstrate that CNVs are a normal feature of the mammalian placental genome, which are regulated systematically during pregnancy.

## Introduction

While the accumulation of somatic copy number variations (CNVs) has been proposed to be a result of the aging process, predisposing cell types to cancer progression and neurological diseases, an alternate hypothesis is that they are a normal—or even essential—part of cell biology [1], [2]. In support of the latter, lymphocyte-specific CNVs in immunologically important genes generate the genetic diversity of receptor molecules critical to their function [3]. Although V(D)J recombination is found only in the immune system, recent reports hint that lineage-specific somatic CNVs may be essential for healthy cellular differentiation and function in a number of organs such as the liver, pancreas and skin [4], [5]. It is unknown how these lineage-specific mammalian CNVs are formed—whether by a process similar to V(D)J recombination or by an alternative mechanism.

Although the role of many cell-type specific CNVs in mammals is unclear, lineage-specific CNVs are a normal aspect of cellular development in the fruit fly *Drosophila melanogaster* [6]. Lineage-specific CNVs form during *Drosophila* egg and larval development in polyploid cells via cycles involving DNA replication in the absence of cell division (endoreplication) [6]. In egg formation, somatic CNVs form by selective amplification of genomic regions containing chorion (eggshell) genes, which facilitates secretion of chorion proteins by the ovarian follicle cells [7], [8]. *Drosophila* somatic CNVs can also arise due to underreplication of certain genomic regions in the salivary glands, fat body and midgut of the larva [9]–[13]. While CNVs in *Drosophila* polyploid cells have been observed for more than 70 years [14], it is not known whether a similar mechanism is present in mammalian cells. However, the recent observation of human tissue-specific CNVs [1]–[5] suggests that somatic CNVs are as essential in mammalian cells as they are in *Drosophila*.

Mammals absolutely require polyploid placental cells, corollaries to *Drosophila* follicle cells, for pregnancy maintenance [15]. In the placenta, polyploidy is restricted to specialized trophoblast cells that invade and remodel the uterus to promote vascularization and other maternal adaptations to pregnancy [15]. In rodents, these cells—termed trophoblast giant cells (TGCs), have 50–1,000 copies of the genome per cell. While proper TGC function depends on their polyploidy content [16], [17], it is not known what aspect of polyploidy is necessary for fetal survival. As TGCs are a class of critical polyploid support cells analogous to *Drosophila* follicle cells, they may similarly use differential replication of the genome to achieve highly specialized function.

Previous studies have addressed possible CNVs in rodent TGCs. Ohgane et al. [18] used restriction landmark genomic scanning (RLGS) to analyze CpG islands in rat junctional zone TGCs during late gestation (days 18 and 20). They reported that ≥97% of the spots detected by RLGS were similar to diploid controls and therefore concluded that there are no TGC CNVs. Sher et al. [19] also argued against the existence of CNVs based on array Comparative Genomics Hybridization (aCGH) and quantitative real-time PCR experiments on mouse e9.5 implantation site TGCs. However, as there are several subtypes of TGCs which all have varying ploidy and functional significance during gestation [15], [20], CNVs could be present in a subset of cell types or only at certain developmental time points. Of particular interest are parietal TGCs, which have the highest degree of polyploidy [15], and are therefore an excellent candidate for differential replication of the polyploid genome. Genetic mouse mutants affecting the parietal TGCs predominantly die before e12.5 [15]–, suggesting that this is when developmentally important CNV would be required.

Here we report that somatic CNVs are a normal part of placental cell biology. We utilized whole genome sequencing (WGS) and aCGH to identify 47 reproducibly underrepresented (UR) domains in mouse e9.5 parietal TGCs, totaling 6% of the genome. Employing a variety of genomic techniques, we demonstrate that UR domains are marked in chromatin prior to endoreplication in TGC progenitor cells and gradually form during the first half of gestation. UR domains are highly enriched for genes involved in cell adhesion and neurogenesis, as well as for gene deserts. Furthermore, we specifically show that UR domains are due to underreplication rather than somatic deletions. Together, these data reveal that lineage-specific CNVs are inherent features of the TGC genome, which are established and regulated throughout placental development.

## Results

### Polyploid TGCs have recurrent and reproducible CNVs

**Figure 1:**
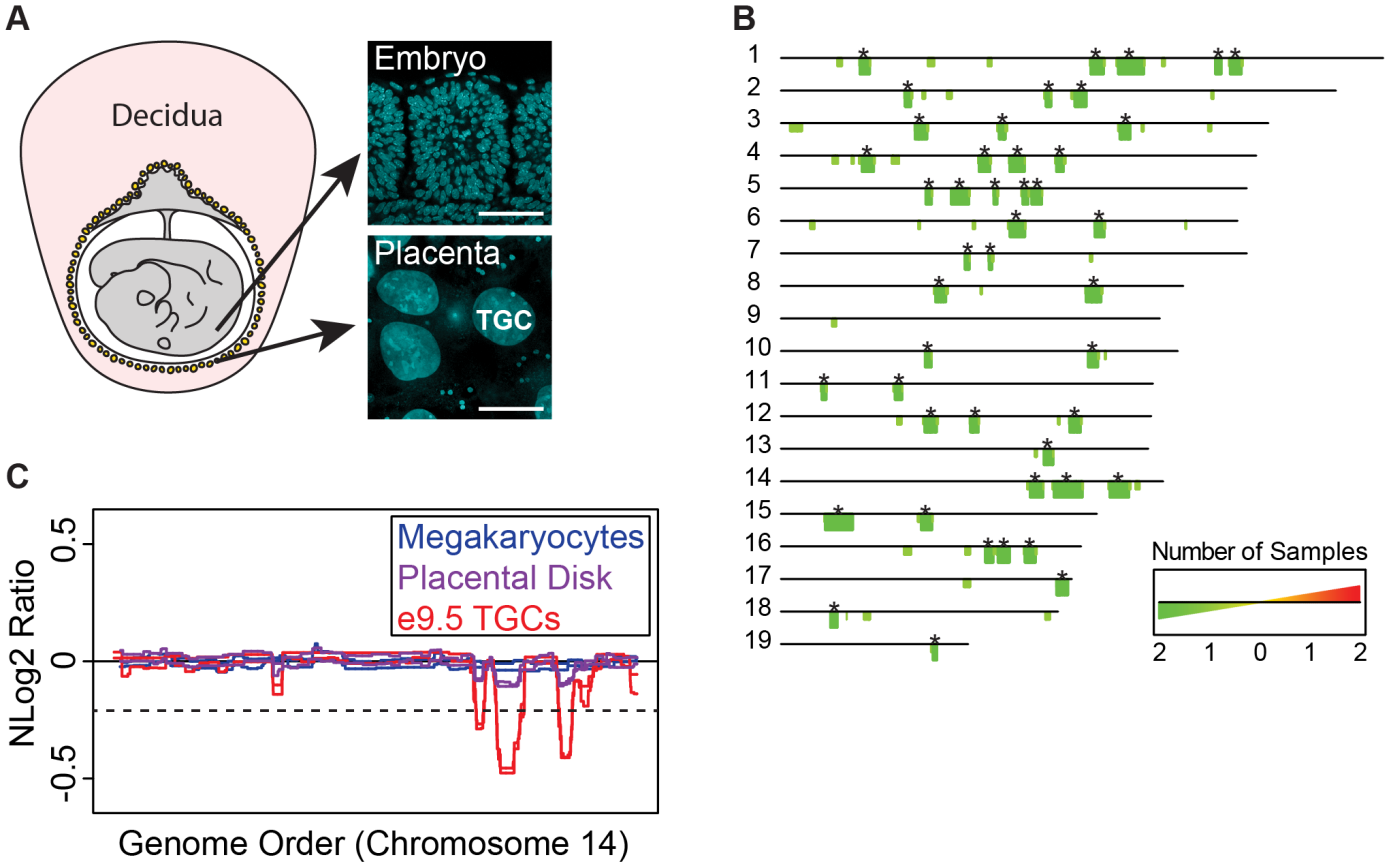
UR domains are specific to TGCs. **A**. TGCs in relationship to other embryonic and maternal tissues at embryonic day 9.5. Left: schematic of e9.5 conceptus. Yellow: parietal TGCs; gray: other embryonic/extraembryonic tissue; pink: maternal decidua. Right: confocal images of TGCs and embryonic cells (somites) stained for DAPI (blue) to show nuclear size. Scale bar = 75 µm. **B**. Location and reproducibility of UR domains on the autosomes of e9.5 TGCs. Summary of results from both biological replicates of e9.5 TGC vs. embryo aCGH (FDR = 0.0001). Darker green/longer bars (asterisks) indicate UR domains present in both replicates. **C**. UR domains are specific to TGCs. Plot comparing position along chromosome 14, a representative chromosome, to the normalized log 2 ratio (NLog2 Ratio) of array intensity of test vs. control. Red: e9.5 TGC vs. embryo; purple: placenta vs. embryo; blue: megakaryocyte vs. embryo. Two biological replicates are plotted for each cell type. Dashed line: FDR = 0.0001. All autosomes shown in Figure S2.

To investigate whether the 50–1,000 genomic copies in polyploid TGCs are uniformly replicated or contain CNVs, we used aCGH to compare genomic regions of mouse parietal TGCs (TGCs) and 2N embryos at e9.5 (Figure 1A, Figure S1A). We dissected four embryos and associated TGCs from one litter, representing pairs of genetically identical tissues, performed aCGH using the Agilent SurePrint G3 Mouse CGH Microarray Kit (two embryos/TGCs pooled per biological replicate), and analyzed the data using the R/Bioconductor package cghFLasso [21]. We identified 45 regions, reproducible between biological replicates, that were underrepresented within the TGC genome compared to the embryonic genome at a false discovery rate (FDR) of 0.0001, which we termed underrepresented (UR) domains (Figure 1B, Table S1). UR domains range in size from 1,037 kb to 9,429 kb (Table S1). In addition to the 45 UR domains common to both replicates, we found 30 domains specific to only one replicate (Figure 1B). However, when we reduced the FDR (to 0.01), 19/30 of these domains are found in both replicates, suggesting that while the degree of underrepresentation varies, UR domains form in specific regions of the genome. Importantly, we did not observe any overrepresented regions in TGCs (FDR = 0.0001).

We next asked whether UR domains were specific to TGCs, or whether they existed in diploid trophoblast cells or other endocycling polyploid cells. We used aCGH to compare the DNA of megakaryocytes (up to 64N) to embryos, placental disk cells (mostly 2N) to embryos, and cultured trophoblast stem cells (TS cells; 2N) to embryonic stem cells (ES cells; Figure 1C, Figure S1B, Figure S2). Megakaryocytes have no detectable underrepresented regions and display one region of overrepresentation common to both replicates, indicating that TGC UR domains are not simply explained by endocycling (FDR = 0.0001; Table S2). Placental disk cells lack any over or underrepresentation (FDR = 0.0001; Table S3), although greatly reducing the FDR (to ≥0.05) revealed a weak trend towards UR domains in the same locations as in TGCs, likely explained by the normal presence of a small number of TGCs within this population (Figure 1C, Figure S2). Finally, we identified several TS and ES specific CNVs, but these were different from the TGC UR domains and presumably represent adaptations to cell culture (Tables S2 & S3) [22]. These data suggest that UR domains are important genomic features unique to TGCs.

As Sher et al. [19] have argued against the existence of CNVs in e9.5 TGCs, we compared our aCGH data to theirs. Consistent with Sher et al., we did not find any CNVs in their data using the R/Bioconductor package cghFLasso and an FDR of 0.0001 [21]. However, greatly reducing the FDR (to >0.05) revealed a trend towards UR domains in the same locations as in our TGC data (Figure S3), similar to the report by Sher et al. of finding reduced copy number using a smaller threshold. Moreover, the Sher et al. data bears a striking resemblance to our placental disk data (Figure S3), suggesting that their study, on implantation site TGCs, is on a population of trophoblast cells more akin to the placental disk than to the parietal TGCs of the mural trophectoderm described in our study. In support of this, while parietal TGCs surround the entire conceptus, TGCs over the central region of the placental disk are smaller and less polyploid than those at the periphery [20]. Together, these data suggest that the parietal TGCs of the mural trophectoderm not only have a higher degree of ploidy, but also have specific CNVs compared to the rest of the placenta.

### Whole genome sequencing reveals UR domains in individuals

**Figure 2:**
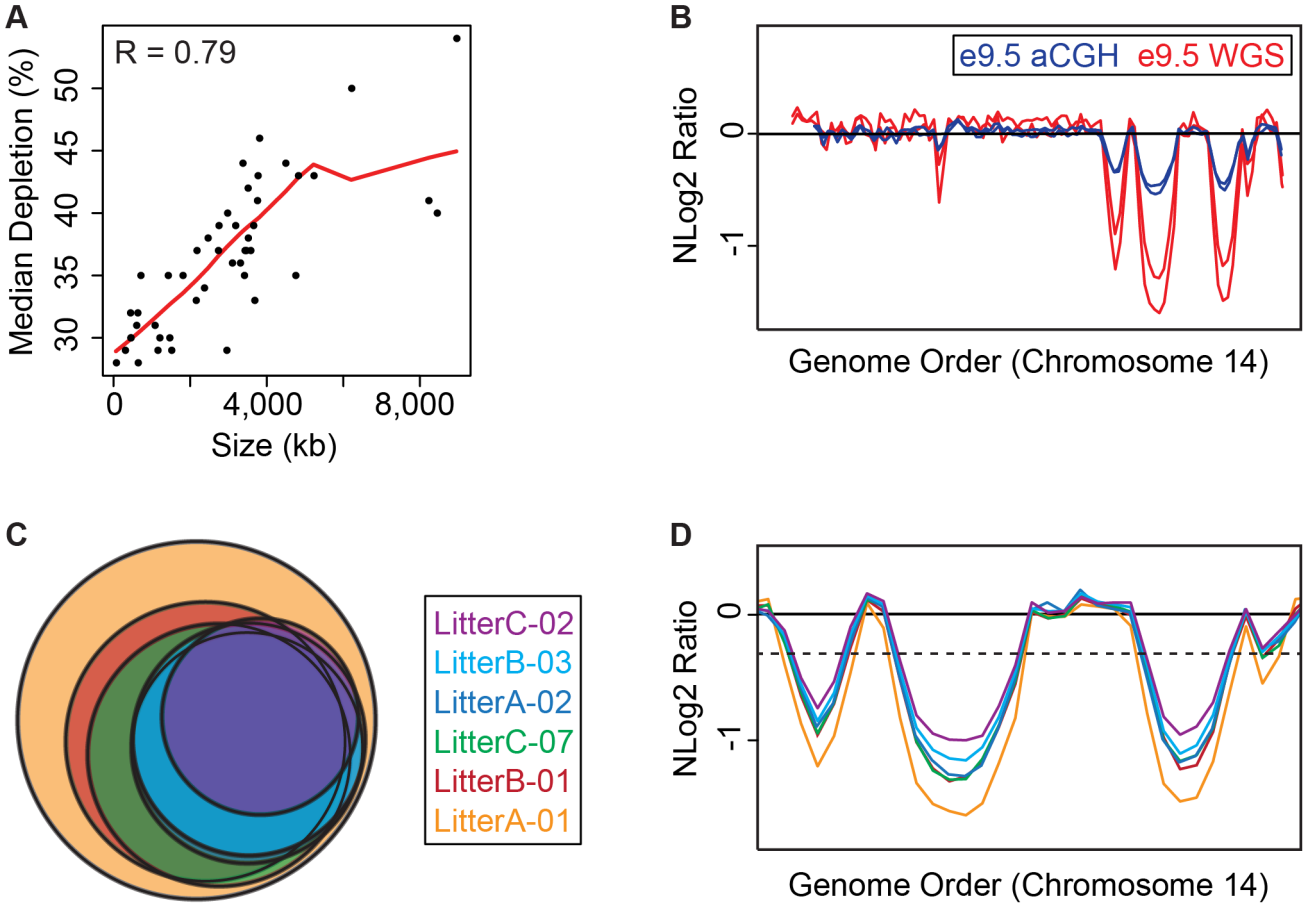
e9.5 UR domains characteristics. **A**. UR domain size and depletion are correlated. Plot of size (0–8,500 kb) versus percent chromosomal median depletion (25–60%) of UR domains. **B**. UR domains are found using two different platforms: aCGH and WGS, although the calculated degree of underrepresentation is increased using WGS. Plot comparing position along chromosome 14 to the NLog2 Ratio of array intensity (aCGH) and sequence coverage (WGS) of TGCs vs. embryos. Red: e9.5 WGS; blue: e9.5 aCGH. Two biological replicates are plotted for each platform (LitterA shown for WGS). All autosomes shown in Figure S4. **C**. Individuals with a lesser number of UR domains have a subset of the UR domains found in the samples with a greater number of UR domains. Venn diagram showing UR domain overlap between six individuals from three separate litters (A, B and C). **D**. UR domains among individuals are nested. Plot comparing position along the last half of chromosome 14 to the NLog2 Ratio of sequence coverage of TGCs vs. embryos. Color code the same as in (**B**). Dashed line: cut-off for significance. All autosomes shown in Figure S5.

To quantitatively examine the extent of underrepresentation in TGCs, we performed paired-end WGS [23]. We sequenced (at 10× coverage) six individual e9.5 TGCs and their genetically matched embryos from three separate litters (2 individuals per litter; Table S4). To identify CNVs, we used a custom R/Bioconductor program based on CNVnator [24], which identifies CNVs at a p-value of 0.01. We found 47 reproducible UR domains on the autosomes in e9.5 TGCs in all samples (Table S5). UR domains range from 75 kb to 8,965 kb and cover 6% of the genome (138 Mbs of 2,717 total Mbs; Table 1). We next calculated the fold depletion of each UR domain from the normalized log 2 ratio of sequence coverage of TGC/embryo [25] and found an average reduction between 27% and 51%, with a median between 28% and 54% (Table 1). Further, the size and degree of depletion of UR domains correlate such that the larger the size of the domain, the greater the degree of underrepresentation (Figure 2A).

**Table 1:** UR domains in e9.5 TGCs.

Chr	Start-Stop	Size (bp)	Ave Mean Log2 (% Depl)	Ave Median Log2 (% Depl)	Genes
1	26,170,000–29,150,000	2,980,000	−0.71 (39%)	−0.73 (40%)	SNORA17, 4931408C20Rik, n-R5s209, Gm11161, Gm7846, Gm597, Gm5697, Gm17684, Gm6462
1	49,295,000–49,740,000**	445,000	−0.53 (31%)	−0.55 (32%)	C230029F24Rik
1	101,895,000–105,555,000	3,660,000	−0.69 (38%)	−0.71 (39%)	SNORA17
1	110,610,000–118,845,000	8,235,000	−0.75 (41%)	−0.77 (41%)	SNORA17, 7SK, Cdh7, Cdh19, Dsel, SNORA68, SNORA42, 9330185C12Rik, Cntnap5a
1	143,185,000–143,900,000	715,000	−0.60 (34%)	−0.62 (35%)	–
1	147,215,000–150,595,000	3,380,000	−0.81 (43%)	−0.84 (44%)	Gm5263, Fam5c, Gm15583, A230059L01Rik, Gm15584, Gm9931, SNORA17
2	41,835,000–42,470,000	635,000	−0.55 (32%)	−0.55 (32%)	Gm13461, Gm13463
2	96,125,000–99,900,000	3,775,000	−0.78 (42%)	−0.81 (43%)	Lrrc4c, 4930445B16Rik, Gm17075, Gm17076, SNORA42, SNORA17, SNORA42, Gm13803, Gm13805, Gm13804, U7, Gm13806, Gm10801*, Gm10800*, Gm13809, Gm13808
3	43,770,000–47,460,000	3,690,000	−0.56 (32%)	−0.57 (33%)	U6, 2610316D01Rik, Pcdh10, U1, SNORA17, Pabpc4l, Gm10356
3	71,440,000–73,815,000	2,375,000	−0.59 (34%)	−0.60 (34%)	U2, Sis, Slitrk3, 7SK, Bche
3	110,690,000–113,795,000	3,105,000	−0.64 (36%)	−0.65 (36%)	SNORA17, Gm5548, Amy2b, Amy2-ps1, Amy2a5, Amy2a4, Amy2a, Amy2a2, Amy2a1, Amy1, Rnpc3*, Col11a1, Dpyd, 7SK
4	26,495,000–29,965,000	3,470,000	−0.67 (37%)	−0.67 (37%)	Gm11904, Gm11902, Gm11901, U6, Gm11914*, Tpm3-rs2*, Gm11911, Gm11907, SNORA17, Gm11908, Gm11909, Gm11910, Gm11912, Gm11913, Epha7, Gm11915, Gm11917, Gm11916, Gm11918, Gm11919, Gm11923
4	37,600,000–38,205,000**	605,000	−0.53 (31%)	−0.54 (31%)	Gm12375, Gm12378, Gm12379, Gm12380
4	65,085,000–68,045,000	2,960,000	−0.49 (29%)	−0.49 (29%)	Astn2, Gm11484, SNORA67, Gm11220, Tlr4, Gm11403, Hmgb1-rs18, Gm11249*, Gm11751, U1, Tcp1-ps1, Gm12911
4	75,100,000–79,600,000	4,500,000	−0.79 (42%)	−0.84 (44%)	Gm11256, Gm11241, Ptprd*, Gm11252, Gm11242, Gm11243, Gm11244, Gm11245, Gm11246, Gm11260, Gm11261, 7SK, Gm11262, Gm12913, SNORA17, Gm11263, Gm11409
4	90,745,000–92,175,000	1,430,000	−0.62 (35%)	−0.63 (35%)	Gm12644, Elavl2, Gm12653, Gm12668, Gm12670, Gm12667, Gm12669, Gm12671, 4930577H14Rik, Izumo3, Gm12666, Gm12638
5	47,605,000–48,815,000	1,210,000	−0.51 (30%)	−0.51 (30%)	Slit2, Mir218-1, Pacrgl*, Kcnip4
5	56,225,000–61,460,000	5,235,000	−0.79 (42%)	−0.81 (43%)	SNORA17, 4932441J04Rik, Gm8121*, Pcdh7, U6, Cbfa2t2-ps1
5	70,235,000–70,310,000	75,000	−0.45 (27%)	−0.47 (28%)	–
5	82,380,000–85,120,000	2,740,000	−0.67 (37%)	−0.67 (37%)	Mir1187, U6, Tecrl, Gm15626, SNORA17, U4, Epha5, Hmgn2-ps1
6	75,110,000–79,870,000	4,760,000	−0.62 (35%)	−0.62 (35%)	Gm9001, U1, Gm9008, U3, Ctnna2, Lrrtm1, Gm5576, Reg3b, Reg3d, Reg3a, Reg2, Reg1, Reg3g
6	102,635,000–106,155,000	3,520,000	−0.65 (36%)	−0.68 (38%)	U6, Chl1, U7, Cntn6, SNORA48, Gm15631, Cntn4
7	60,935,000–61,390,000	455,000	−0.51 (30%)	−0.51 (30%)	–
8	100,035,000–104,865,000	4,830,000	−0.78 (42%)	−0.82 (43%)	U6atac, Gm15679, Cdh8, U6, Gm15681, Gm15680, A330008L17Rik, Gm8688, Gm15210*, Gm5742, Gm3662, Gm17316, SNORA17
8	50,675,000–54,110,000	3,435,000	−0.67 (37%)	−0.66 (37%)	U7, U1, Gm9892*, U2, SNORA17
10	47,750,000–48,835,000	1,085,000	−0.54 (31%)	−0.54 (31%)	SNORA25, U4, Grik2
10	100,945,000–103,700,000	2,755,000	−0.72 (39%)	−0.72 (39%)	SNORA17, Mgat4c, Nts, Rassf9, Alx1, Gm17028, Lrriq1, Slc6a15, U6, Gm6763, Gm8766, Gm4340, U7
11	13,635,000–13,945,000	310,000	−0.48 (28%)	−0.49 (29%)	4930554G24Rik
11	37,290,000–39,450,000	2,160,000	−0.58 (33%)	−0.58 (33%)	Gm12128, Gm12129, Gm12130, U6
12	47,225,000–50,645,000	3,420,000	−0.62 (35%)	−0.62 (35%)	Nova1, 7SK, U4, Gm1818, U6, Foxg1, 3110039M20Rik, Gm9804
12	62,165,000–64,635,000	2,470,000	−0.68 (38%)	−0.70 (38%)	Lrfn5, 7SK, U6, Spanxn4, Gm5185
12	94,500,000–98,315,000	3,815,000	−0.84 (44%)	−0.90 (46%)	Gm9726, U7, U6, Flrt2, 1700019M22Rik, 7SK, Gm6863*
13	86,650,000–88,830,000	2,180,000	−0.67 (37%)	−0.66 (37%)	Gm8526
14	81,615,000–85,375,000	3,760,000	−0.74 (40%)	−0.76 (41%)	U1, snR78, SNORA17, Pcdh17
14	89,430,000–98,395,000	8,965,000	−1.04 (51%)	−1.12 (54%)	U6, SNORA17, Gm10110*, Pcdh9, U1, 4921530L21Rik, Klhl1, Gm15515, U5, SNORA30, U7, Dach1
14	107,745,000–113,965,000	6,220,000	−0.95 (48%)	−1.01 (50%)	SNORA17, Rpl27a-ps2, Slitrk1, n-R5s50, U6, Slitrk6, Gm6280, Slitrk5, Gm4822, Tpm3-rs7*, Gm4487
15	14,605,000–23,060,000	8,455,000	−0.71 (39%)	−0.73 (40%)	SNORA17, Cdh9, 7SK, U6, C030047K22Rik, Cdh10, Acot10, Cdh12, Cdh18, Gm5803, Cdh18
15	45,850,000–49,435,000	3,585,000	−0.66 (37%)	−0.67 (37%)	U6, 4930548G14Rik, U7, Csmd3, Gm16300, SNORA17, U2
16	40,820,000–41,465,000**	645,000	−0.48 (28%)	−0.48 (28%)	–
16	67,275,000–69,090,000	1,815,000	−0.62 (35%)	−0.62 (35%)	Gm15828, SNORA17, 7SK
16	71,335,000–74,650,000	3,315,000	−0.63 (35%)	−0.64 (36%)	SNORA71, Robo1, U1, Robo2, SNORA70, SNORA17
16	79,945,000–83,460,000	3,515,000	−0.75 (41%)	−0.79 (42%)	SNORA17, U6, Ncam2, 7SK, U6, Rpl21-ps5
17	90,665,000–93,855,000	3,190,000	−0.71 (39%)	−0.71 (39%)	Gm10493, 4930480K15Rik, Gm10308, Gm6741, Gm15404, Gm15403, SNORA17, Gm15405, Adcyap1, U6
18	16,755,000–18,275,000	1,520,000	−0.51 (30%)	−0.50 (29%)	Gm15328, Gm7670, 1700001G01Rik, SNORA17, 7SK
18	27,570,000–29,040,000**	1,470,000	−0.51 (30%)	−0.51 (30%)	Gm7729, SNORA17
18	86,920,000–87,375,000**	455,000	−0.51 (30%)	−0.51 (30%)	–
19	50,135,000–51,295,000	1,160,000	−0.49 (29%)	−0.50 (29%)	Rpl13a-ps1*, Sorcs1, Gm16745, 5S_rRNA

UR domain location, size, depletion, and genetic content based on six e.5 WGS individuals. Genes are called from Ensembl. Asterisks mark the 15, out of 316, genes that have low-level expression in UR domains. Double asterisks mark UR domains that are not found in aCGH data unless a less stringent FDR (of 0.01) is used.

Next, we examined how much variation existed between individuals. First, we compared aCGH and WGS data, and found 43 UR domains common to both platforms (Figure 2B, Table 1, Figure S4, Table S1). Of the domains that differ, five additional domains in the WGS data are likely due to the greater sensitivity of WGS, as these domains can also be found in the aCGH data if the FDR is lowered (to 0.01). Three additional domains in the aCGH data are found in a majority of the WGS samples (present in four to five out of the six samples), suggesting a small amount of variability in UR domain formation (Tables S1 & S5). To examine this variability in more depth, we examined the six individual WGS samples. Besides the 47 UR domains common to all six samples, we also found underrepresented regions present in only a subset (Figure 2C, Figure S5, Table S5). In general, samples with the least number of UR domains have a subset of the domains found in the samples with the most (Figure 2C, Figure S5, Table S5). In addition, the size of a particular UR domain is generally smaller in samples with fewer UR domains (Figure 2D, Table S5). As the samples vary slightly in age, this suggests that UR domains amass over time, such that slightly younger placentas have fewer and smaller UR domains.

### The number, size and degree of depletion of UR domains expands during early gestation

**Figure 3:**
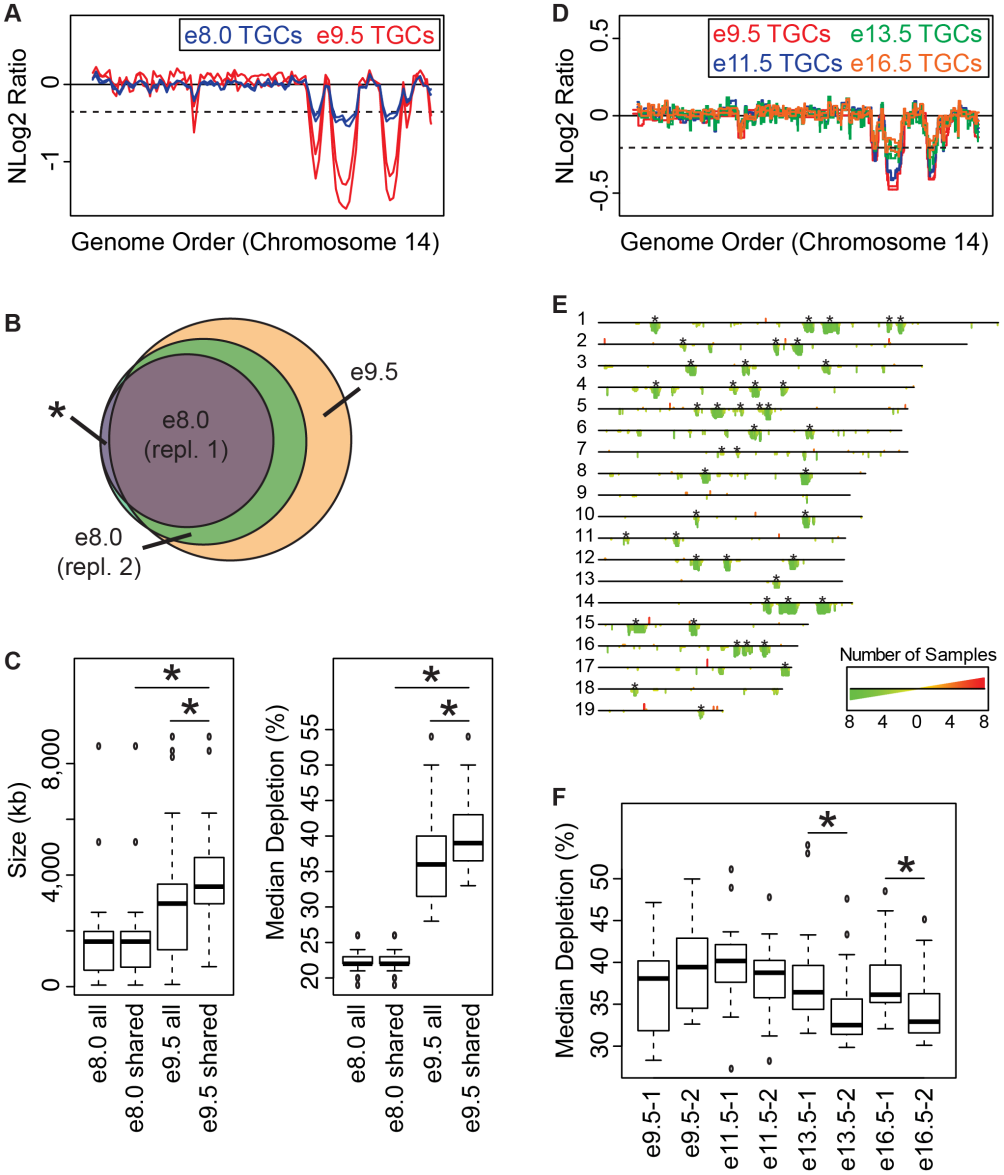
Degree of underrepresentation varies over developmental time. **A**. UR domains exist at e8.0 and further develop over time. Plot comparing position along chromosome 14 to the NLog2 Ratio of sequence coverage of TGCs vs. embryos. Red: e9.5; blue: e8.0. Two biological replicates are plotted for each stage (LitterA shown for WGS). Dashed line: cut-off for significance. All autosomes shown in Figure S6. **B**. e8.0 TGCs have a subset of the UR domains found at e9.5. Venn diagram showing overlap of UR domains between both e8.0 replicates (one litter each) and UR domains common to all six e9.5 individuals. Asterisk represents the one UR domain present in both e8.0 replicates that is present in only 5/6 of the e9.5 individuals. **C**. Size and depletion of UR domains increases between e8.0 and e9.5. “e8.0 all”: UR domains present in both e8.0 replicates; “e8.0 shared”: UR domains present at e8.0 that are also present at e9.5; “e9.5 all”: all UR domains present in all six e9.5 individuals; “e9.5 shared”: UR domains present at e9.5 that are also present at e8.0. Box plot on left compares these classes of UR domains to size (0–8,500 kb), while box plot on right compares these classes to percent median depletion (15–60%). Asterisks mark comparisons that are statistically significant (p<0.01). **D**. UR domains present at late gestation. Plot comparing position along chromosome 14 to the NLog2 Ratio of array intensity of TGC vs. embryo. Red: e9.5; blue: e11.5; green: e13.5; orange: e16.5. Two biological replicates are plotted for each stage. Dashed line: FDR = 0.0001. All autosomes shown in Figure S7. **E**. Location of UR domains during the second half of gestation. Summary of results from both biological replicates of aCGH of TGCs from e9.5, e11.5, e13.5, and e16.5, all versus embryos (FDR = 0.0001). Darker green/longer bars indicate UR domains present in more replicates. Asterisks indicate the location of UR domains present at e9.5. **F**. Depletion of UR domains does not significantly change between e9.5 and e16.5, however, depletion of UR domains significantly differs between biological replicates at e13.5 and e16.5. Box plot compares percent median depletion of each biological replicate at stages e9.5, e11.5, e13.5, and e16.5. To compare with (**C**), aCGH data was normalized to WGS depletion levels (e9.5). Asterisks mark comparisons that are statistically significant (p<0.01).

To test our hypothesis that UR domains develop over time, we performed WGS on e8.0 TGCs/embryos (one litter per replicate) and compared these results to e9.5. We found 24 domains common to both biological replicates at e8.0, versus 47 domains common to all samples at e9.5 (Figure 3A & 3B, Figure S6). All e9.5 individuals have 23 of these domains with 5/6 individuals containing the remaining domain (Figure 3B). We also found 10 domains unique to one of the two biological replicates at e8.0; 10/10 of these domains are contained in all e9.5 individuals (Figure 3B). Finally, we found that both size and degree of depletion of UR domains significantly increase between e8.0 and e9.5 (Figure 3C). Overall, as all UR domains at e9.5 are also present at e8.0, and UR domains at e9.5 are also more numerous, larger and more depleted, we propose that they are gradually established during early gestation.

### New small and stochastic CNVs form in later gestation

We next asked whether the number and degree of depletion of UR domains continues to increases throughout development. We performed aCGH on TGCs/embryos collected from the second half of gestation—e11.5, e13.5, e16.5—and compared them to e9.5. Out of 45 UR domains present in both biological replicates at e9.5 (FDR = 0.0001), 22 of these are present in all biological replicates at e11.5, e13.5 and e16.5, and an additional 10 (32/45) are present in all samples except for one of the e16.5 replicates (Figure 3D & 3E, Figure S7). We next examined size, and found that the 32 common domains are significantly larger than UR domains that arise later in development (the 147 not present at e9.5; Figure 3D & 3E, Figure S7). However, unlike between e8.0 and e9.5, where the degree of depletion expanded, we found no significant change from e9.5 to e16.5 (Figure 3F). Although, UR domains slightly trend towards becoming less depleted over time (Figure 3D & 3F, Figure S7). There is also more intrinsic variability later in gestation, as the median degree of depletion between biological replicates at both e13.5 and e16.5 is significantly different (Figure 3F). The differences between UR domains in early (e8.0–e9.0) and later (e11.5–e16.5) gestation correlate with previous data showing that TGC polyploidy drastically increases until e10.5, and endocycling ends by e13.5 [20]. These data suggest that the increase in UR domain size and degree of underrepresentation from e8.0 to e9.5 is linked to the robust endocycles of early gestation. Furthermore, the termination of endocycles in later development may free cellular machinery to increase representation levels in UR domains.

We also found 33 overrepresented regions at e11.5–e16.5 that are not present at e9.5 (Figure 3D & 3E, Figure S7). We examined gene content of overrepresented regions common to at least two staged biological replicates (10/33), but did not find any annotated genes. Thus, while new CNV regions form during late gestation, they are more stochastic, less reproducible, and significantly smaller than those conserved between all stages.

### UR domains form during *in vitro* differentiation

**Figure 4:**
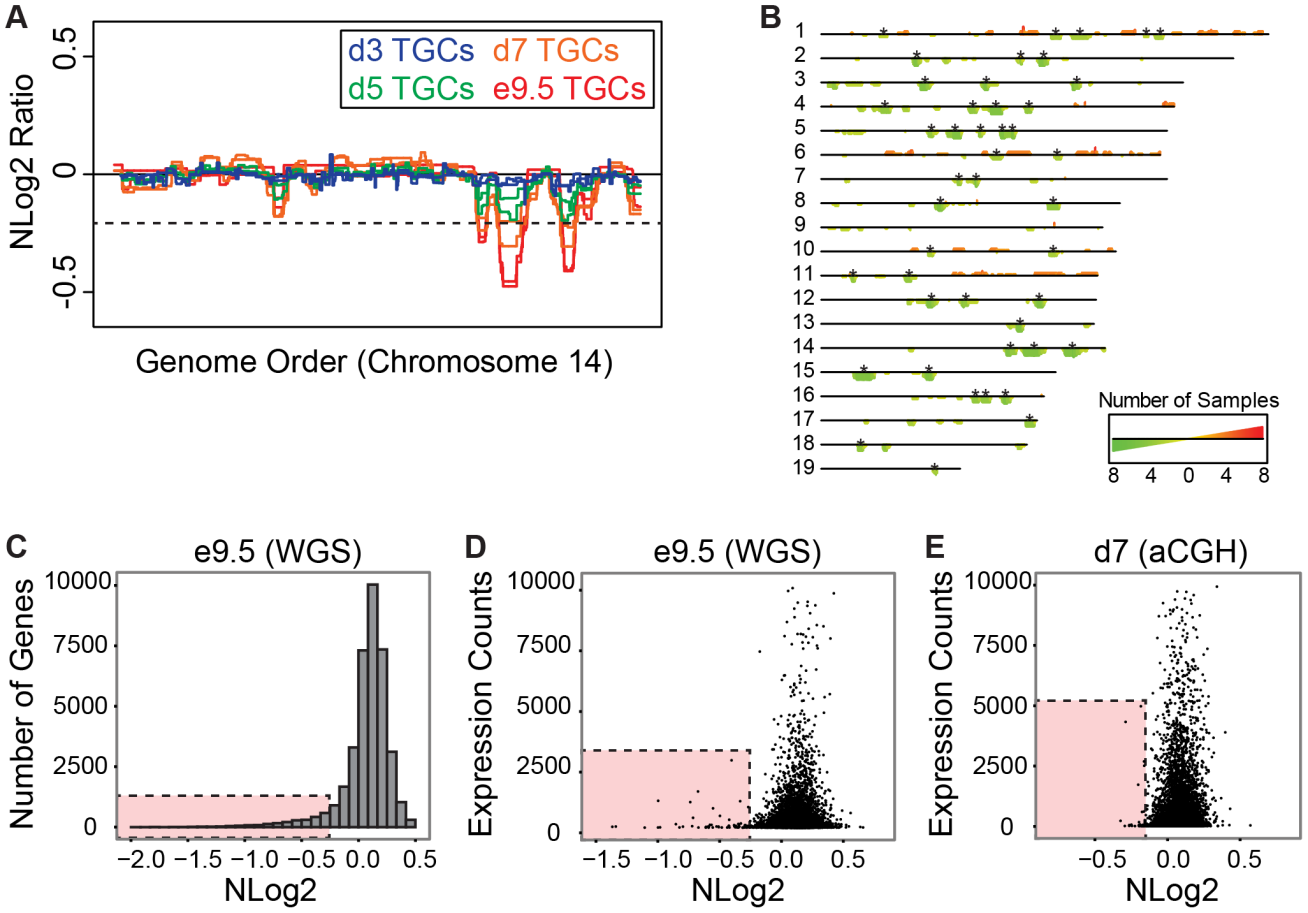
UR domains have low gene content and expression both *in vivo* and *in vitro*. **A**. *In vitro* TGCs produce the same UR domains as *in vivo*. Plot comparing position along chromosome 14 to the NLog2 Ratio of array intensity of TGC vs. embryo (e9.5) and TGC vs. TS cells (day 3, 5, and 7). Red: e9.5 (*in vivo*); blue: day 3 (*in vitro*); green: day 5 (*in vitro*); orange: day 7 (*in vitro*). Two biological replicates are plotted for each cell type. Dashed line: FDR = 0.0001. All autosomes shown in Figure S8. **B**. Location of UR domains on the autosomes of cultured TGCs compared to e9.5 *in vivo* TGCs. Summary of results from both biological replicates of aCGH of cultured TGCs differentiated 3, 5 and 7 days vs. TS cells, and of e9.5 *in vivo* TGCs vs. embryo (FDR = 0.0001). Darker green/longer bars indicate UR domains present in more replicates. Asterisks indicate the location of UR domains present in both replicates at e9.5. **C**. UR domains are gene poor. Histogram plotting number of Ensembl genes versus level of representation (NLog2 of TGCs vs. embryos (WGS)). UR domains boxed in pink. **D**. Low gene expression in UR domains *in vivo*. Plot of TGC normalized expression (NE) counts versus level of representation (NLog2 of e9.5 TGCs vs. embryos (WGS)). UR domains boxed in pink. **E**. Low gene expression in UR domains *in vitro*. Plot of TGC NE counts versus level of representation (NLog2 of day 7 TGCs vs. TS cells (aCGH)). Genes not present on the array were filtered out. UR domains boxed in pink.

We next examined whether UR domains are also generated *in vitro* when differentiating TS cells into TGCs. To this end, we performed aCGH on purified TGCs harvested at 3, 5 and 7 days after differentiation [26]–[28] (Figure S8). Similar to *in vivo*, *in vitro* cells generate the same UR domains and also develop these over time (FDR = 0.0001, Figure 4A & 4B, Figure S8). At day 3, only one biological replicate has any of the UR domains found *in vivo* at e9.5 (3/45). At day 5, both replicates contain 1/45 domains, and one replicate contains 21/45 domains. At day 7, both replicates contain 34/45 UR domains, and one replicate contains 43/45 domains. Remarkably, *in vitro* cells generate the same UR domains as their *in vivo* counterparts (Figure 4A & 4B, Figure S8), strongly suggesting that the formation of these UR domains is a fundamental feature of TGC development.

### UR domains are highly enriched for genes involved in cell adhesion and neurogenesis

Next, we asked whether genes contained within e9.5 TGC UR domains were enriched for certain biological functions. We found that UR domains are significantly depleted of both protein-coding and non-coding genes as expected by chance (386 observed vs. 617 expected, 0.63× enrichment, p<0.001) and when compared to the rest of the genome (Figure 4C). Further, these domains are significantly enriched for 1 Mb gene deserts (regions without any Ensembl annotations; 47 observed vs. 9 expected, 4.96× enrichment, p<0.001). In total, 386 genes are present within UR domains, 106 of which are functionally annotated. When we examined these 106 genes for function using GOTERMFINDER [29], the top enrichment categories are biological adhesion (p = 2.31×10^−9^) and related categories, followed by neuron projection development (p = 4.23×10^−8^), and related neurogenesis categories. These categories were not enriched when we performed the same analyses on a list of genes found in a random set of regions that have the same length and chromosome distribution. Finally, using 3′ RNA-Seq (3SEQ) [30] from both *in vivo* and *in vitro* TGCs, we compared expression of the genes to the degree of representation and found that genes in UR domains are either not expressed or have much lower levels of transcription than genes in regularly represented regions (Figure 4D & 4E). Overall, our data show that there are specific classes of genes enriched within the UR domains and these genes are generally not expressed, raising the possibility that UR domains function to limit the expression of a particular subset of genes in TGCs.

### UR domains are heterochromatic

**Figure 5:**
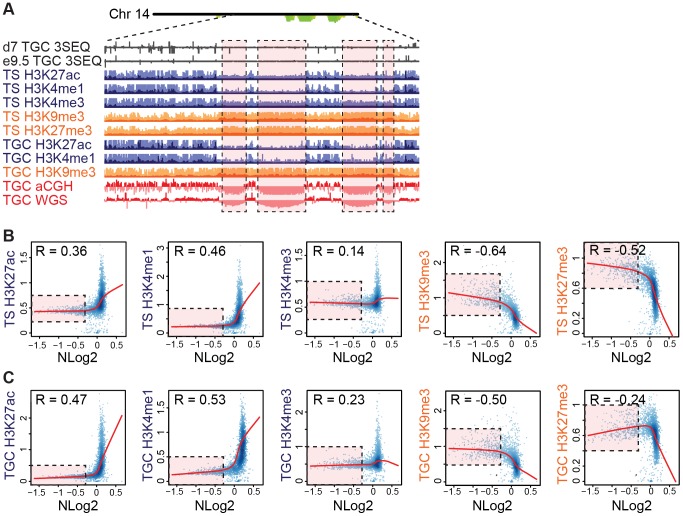
UR domains are heterochromatic. **A**. UR domains correlate with histone marks. Screen shot from the UCSC genome browser of the last half of chromosome 14 (schematic shown above screen shot) showing the following: 3SEQ from *in vivo* e9.5 TGCs and *in vitro* d7 TGCs (black), active histone marks (H3K27ac, H3K4me1, H3K4me3; dark purple) and repressive histone marks (H3K9me3, H3K27me3; orange) for cultured TS cells and TGCs, and aCGH and WGS data from *in vivo* e9.5 TGCs (pink/red). Histone mark mean is darker color, maximum is lighter color. UR domains boxed in pink. **B**. UR domains are a subset of heterochromatin in TS cells. The Pearson correlation (R) between NLog2 values of TGC vs. embryo (WGS) and fold enrichment (FE) for histone marks. Data points represent 1 Mb windows in the genome. UR domains (negative NLog2 values) are correlated with high values for repressive histone marks (negative R values). UR domains (negative NLog2 values) are negatively correlated with high values for active histone marks (positive R values). Red lines represent the lowess line (locally weighted scatterplot smoothing) of the data points. UR domains boxed in pink. **C**. UR domains are a subset of heterochromatin in TGCs. See (**B**) for plot details.

To test whether UR domains are characterized by a specific chromatin state, we performed ChIP-Seq using anti-H3K27ac, anti-H3K4me1, anti-H3K4me3, anti-H3K9me3, and anti-H3K27me3 in both *in vitro* TS cells and derived TGCs [31]. We used MACS2 to determine the normalized fold change for histone occupancy [32] and then used the Pearson correlation (R) to determine how the degree of representation (normalized log 2 of e9.5 WGS) correlates with signals from histone marks. In both TGCs and TS cells, we find that UR domains tend to co-localize with the repressive marks H3K9me3 and H3K27me3 (Figure 5). Conversely, UR domains have underrepresentation of the active chromatin marks H3K4me3, H3K4me1 and H3K27ac (Figure 5). These results demonstrate that UR domains do not occur in active regions of the genome and that they are marked in the 2N progenitor cells (TS cells). Interestingly, UR domains are only a fraction of genomic heterochromatin (Figure 5B & 5C). All UR domains have increased signals for repressive histone marks and only weak signals for active histone marks. However, not all regions of the genome having repressive marks but not active marks are associated with a UR domain. Overall, this demonstrates that UR domains have a heterochromatic signature, but represent only a subset of heterochromatin.

We further examined the relationship between UR domains and heterochromatin using an alternative statistical method. We asked whether the histone marks are significantly enriched or depleted in our defined list of UR domains compared to what would be expected by chance [31]. Similar to our correlation analysis, marks associated with transcriptional activation (H3K4me3, H3K4me1 and H3K27ac) are significantly depleted in UR domains (p<0.001; Table 2). Conversely, the repressive mark H3K9me3 is enriched within UR domains (p<0.001; Table 2). Interestingly, while the repressive mark H3K27me3 is also enriched within UR domains in TS cells, it is depleted within UR domains in TGCs (p<0.001; Table 2). This observation agrees with previous data where extraembryonic cells have lower levels of H3K27me3 methylation than embryonic cells [33], and suggests that H3k27me3 is not critical for UR domain maintenance. Together, our data show that UR domains have a heterochromatic signature, both in TGCs and in their 2N progenitors.

**Table 2:** UR domains are heterochromatic.

TS or TGC	Histone Mark	Active or Repressive	Observed vs. Expected	Enrichment
TS	H3K4me3	Active	291 vs. 473	0.62×
TGC	H3K4me3	Active	715 vs. 904	0.79×
TS	H3K4me1	Active	182 vs. 3745	0.05×
TGC	H3K4me1	Active	115 vs. 2588	0.04×
TS	H3K27ac	Active	149 vs. 1516	0.10×
TGC	H3K27ac	Active	67 vs. 1826	0.04×
TS	H3K9me3	Repressive	13183 vs. 9122	1.45×
TGC	H3K9me3	Repressive	6609 vs. 5949	1.11×
TS	H3K27me3	Repressive	3575 vs. 2631	1.36×
TGC	H3K27me3	Repressive	1143 vs. 1866	0.61×*

UR domains are enriched for repressive histone marks and depleted of active histone marks compared to what is expected by chance. Interestingly, while UR domains in TS cells are enriched for both the repressive mark H3K9me3 and H3K27me3, UR domains in TGCs are only enriched for the repressive mark H3K9me3, and are depleted of the repressive mark H2K27me3 (asterisk). The p-value for all enrichment/depletion values is <0.001.

### UR domains are not caused by deletions

To examine whether UR domains are caused by genomic deletions, we carried out somatic structural variant analysis using paired-end sequencing data from the six TGC and matched embryo samples with the program SMASH [34]. If UR domains are caused by acquired genomic deletions, we would expect to find multiple library inserts that fully span the deleted regions (“discordant” paired-end reads; Figure S9). While we did detect sample-specific CNVs, we did not detect somatic deletions common to all of the six TGCs, but not the embryos. Moreover, the probability of not detecting a given deletion in each of the six samples is extremely low (p = 2•×10^−5^). These data show that UR domains are not a result of somatic chromosomal deletions.

### UR domains are late-replicating chromosomal segments

**Figure 6:**
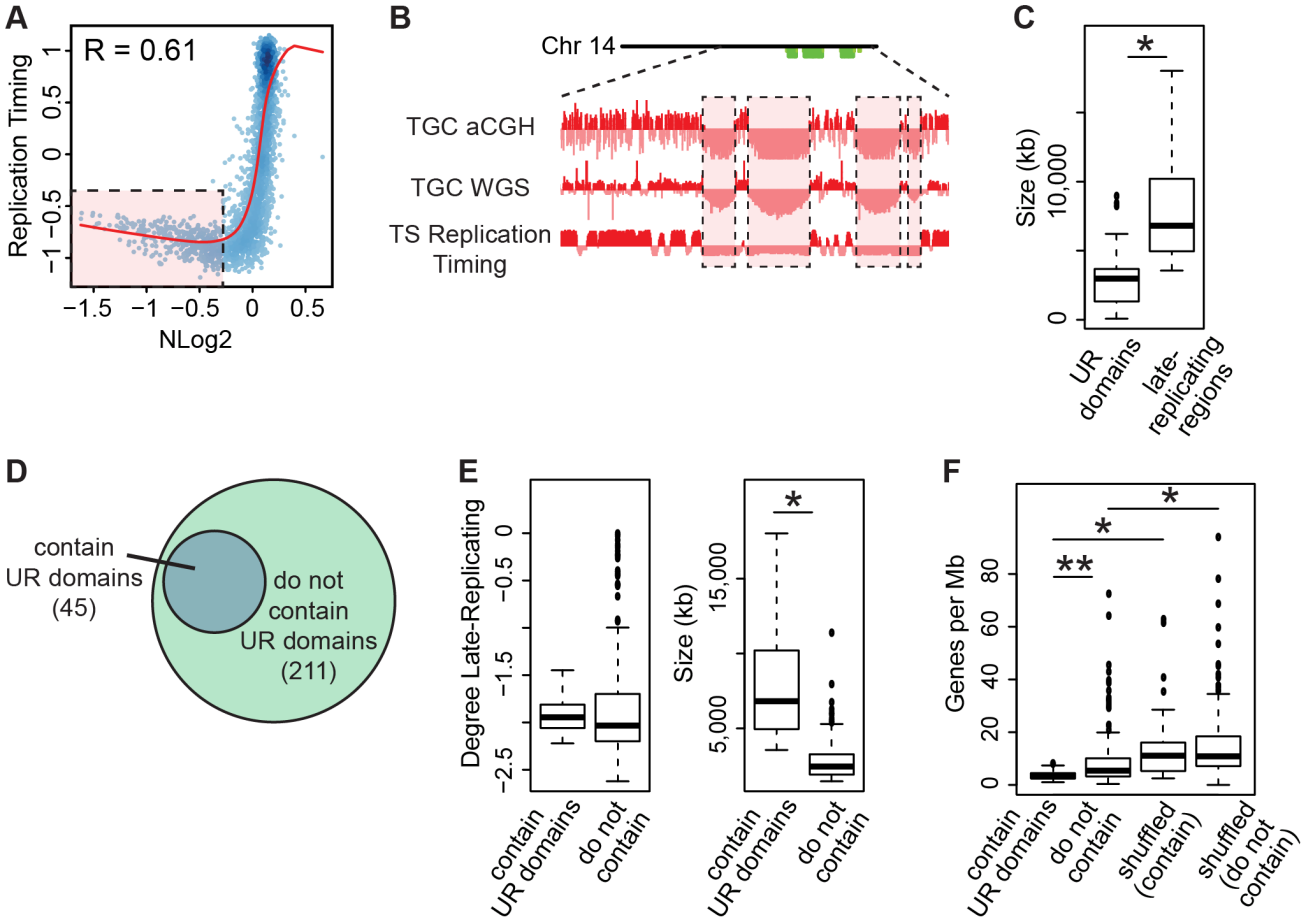
TGC UR domains are a subset of late-replicating regions in TS cells. **A**. UR domains correlate with late-replicating regions in TS cells. The Pearson correlation (R) between NLog2 values of TGC vs. embryo (WGS) and average TS replication timing. Data points represent 1 Mb windows in the genome. UR domains boxed in pink. **B**. UR domains are late-replicating. Screen shot from the UCSC genome browser of the second half of chromosome 14 (schematic shown above screen shot) depicting the following: aCGH and WGS data from e9.5 TGCs and replication timing data from cultured TS cells. UR domains boxed in pink. **C**. Box plot analysis shows that UR domains are smaller than the late-replicating regions that contain them. Asterisk marks the comparison that is statistically significant (p<0.01). **D**. UR domains form from a subset of late-replicating regions. Diagram depicting late-replicating regions that contain UR domains versus ones that do not contain UR domains. **E**. Box plot analysis shows that the late-replicating regions that contain UR domains are significantly larger, but not significantly more late-replicating, than those that do not. Asterisk marks the comparison that is statistically significant (p<0.01). **F**. Box plot analysis shows that the late-replicating regions that contain UR domains have significantly fewer genes than those that do not (double asterisks). “Shuffled” refers to a random set of regions that have the same length and chromosome distribution. Asterisks mark comparisons that are statistically significant (p<0.01).

Since our WGS data does not support genomic deletions as the source of UR domains, we investigated whether they may be due to underreplication (Figure S9B). In 2N cells, replication timing is precisely regulated such that specific regions of the genome are replicated early in S phase while others are replicated late in S phase [35]. To test whether UR domain formation is caused by incomplete replication of regions that are normally replicated late in 2N TS cells, we first generated a replication timing profile of TS cells. To this end, we captured early- and late-replicating regions in TS cells by pulsing an asynchronous cell culture with BrdU to label replicating DNA followed by FACS, and then used aCGH to compare early and late BrdU-containing DNA [36]. Next, we compared late-replicating regions in TS cells to UR domains. Using the Pearson correlation (R), we found that UR domains correlate with late replication (Figure 6A). Also, 47/47 TGC UR domains reside within late-replicating regions in TS cells (Figure 6B, Table S6). UR domains are significantly smaller than the late-replicating regions that they are nested in (Figure 6C; Table S6), suggesting that they are a subset of these larger regions.

Finally, as only 45 of the 211 late-replicating regions contain a UR domain (Figure 6D, Table S6), we asked what distinguishes the late-replicating regions that form UR domains from those that do not. While there is no significant difference in the degree of late replication between these classes, late-replicating regions that contain UR domains are significantly larger (Figure 6E). However, size is not the sole characteristic determining where UR domains form, as not all regions greater than a certain size contain a UR domain. We next investigated gene content and found that late-replicating regions that contain UR domains also contain significantly fewer genes than those that do not (Figure 6F). These regions are also preferentially enriched for 1 Mb gene deserts (58 observed vs. 18 expected, 3.16× enrichment, p<0.001). Together, our data show that UR domains form from a specific class of late-replicating, heterochromatic regions with low gene content, suggesting that UR domains are not simply a byproduct of late-replicating heterochromatin, but are a precisely regulated subset.

## Discussion

We report here the first mammalian example, outside of the immune system, of lineage-specific CNVs being an integral part of normal cell biology and development. Notably, we show that CNVs in placental cells form via a novel mechanism unrelated to V(D)J recombination. Using both aCGH and high-throughput WGS, we identified 47 reproducible underrepresented domains in mouse parietal TGCs totaling 138 Mbs, or 6% of the genome. We found that UR domains are highly enriched for genes involved in cell adhesion and neurogenesis, as well as for gene deserts. Furthermore, we specifically show that UR domains are due to underreplication of a specialized type of heterochromatin, rather than acquired genomic deletions. Our data reveal that lineage-specific CNVs are a normal aspect of the TGC genome that are established and regulated during gestation.

### Establishment of UR domains may involve a novel chromatin remodeler

Only a subset of heterochromatic, late-replicating regions form UR domains, suggesting that UR domains are not simply a byproduct of late-replicating heterochromatin, but are precisely regulated. We propose that either this is dictated by genomic structure or that there are specific DNA binding proteins that define UR domains. We favor the latter model based on parallels found in *Drosophila*, whereby mutants for *Suppressor of Underreplication* (*SuUR*) have underreplicated domains that become replicated to normal levels [12], [13], [37]. However, SUUR protein does not appear to be present in species outside the Drosophilids, and we have not found any *SuUR* homologs in mice via BLAST, raising the possibility that presently unknown proteins in mammals may be regulating this process.

### Lineage-specific CNVs in mammalian development

Lineage-specific CNVs are an overlooked aspect of the mammalian genome. Although recent data suggests that they are widespread [1]–[5], their identification and functional study has not been carried out systematically. Identification of CNVs may be particularly difficult to define in primary tissues, due to high background of cells lacking CNVs. In support of this, Abyzov et al. [4] found a low frequency of somatic CNV in human fibroblasts. Further, even in more homogenous populations, relatively small degrees of CNV may mask their presence. Van Heesch et al. [38] found tissue-specific CNVs in rat blood, brain, liver and testis, where the degree of underrepresentation does not exceed 50%. While Van Heesch et al. conclude that their findings were the result of systematic bias in DNA isolation procedures, they could never get rid of these CNVs using any analytical or experimental approach. Moreover, Manukjan et al. [39] suggest that Van Heesch et al. are identifying the signature of replication timing in their CNV analyses due to the use of proliferating cells. Intriguingly, this suggests that, analogous to polyploid TGCs in the placenta, underreplication may be crucial in organs containing a highly proliferative population of 2N cells.

### Convergent evolution of CNVs in flies and mice suggests function

While CNVs in *Drosophila* polyploid cells have been characterized for more than 70 years [14], our work demonstrates for the first time that CNVs are a normal aspect of mammalian development. The rarity of endoreplicating polyploid cells in animals suggests that CNVs in mouse and *Drosophila* arose independently [6], and therefore may have species-specific differences. While *Drosophila* CNVs are typically 90% underrepresented, mouse CNVs are never more than 50%. We strongly suggest that there are UR domains in both mouse and *Drosophila* polyploid cells, and that the presence of these domains in both taxa is an example of convergent evolution due to similar selective pressures, indicative of functional importance. As both mice and flies have a fast rate of early development compared to related species, formation of UR domains could be an integral part of accelerating the cell cycle, and therefore be a key mechanism behind their rapid life cycles.

### UR domains as a mechanism to drive TGC function

UR domains are a unique feature of the TGC genome, suggesting that they play a central role in placental function and pregnancy. Consistent with this, UR domains are enriched for specific classes of genes involved in cell adhesion and neurogenesis. Intriguingly, there is evidence that downregulation of both classes of proteins is crucial for placental function. Downregulation of cell adhesion genes is necessary for trophoblast invasion in both mice and humans [40], [41]. Further—and quite remarkably—Liao et al. [42] found that upregulation of genes in the SLIT/ROBO neuronal guidance system in the human placenta is associated with the pregnancy disease pre-eclampsia. UR domain formation could also enable TGCs to simply save materials and time, a hypothesis that has been proposed for polyploidy in general [43]. TGCs are essential during the first half of gestation, when it is absolutely critical for the rapidly growing embryo to establish a connection with the mother [15], [44]. Formation of UR domains could allow for more rapid maturation of TGCs by allowing replication initiation to proceed without waiting for replication of nonessential regions of the genome. In support of this, UR domains represent a significant part of the genome, 6% (138 Mbs of 2,717 total Mbs), and therefore the cell would require considerable resources to fully replicate these regions. Together, functional evidence and convergent evolution suggest that UR domains are a critical element during pregnancy. Regardless, placental UR domains are the first mammalian example, outside of the immune system, of lineage-specific CNVs being an integral part of normal cell biology and development.

## Materials and Methods

### Ethics statement

All animal work has been conducted according to relevant U.S. and international guidelines. Specifically, all experimental procedures were carried out in accordance with the Administrative Panel on Laboratory Animal Care (APLAC) protocol and the institutional guidelines set by the Veterinary Service Center at Stanford University (Animal Welfare Assurance A3213-01 and USDA License 93-R-0004). Stanford APLAC and institutional guidelines are in compliance with the U.S. Public Health Service Policy on Humane Care and Use of Laboratory Animals. The Stanford APLAC approved the animal protocol associated with the work described in this publication.

### Mice

129-Elite, C57BL/6 and pregnant C57BL/6 mice were obtained from Charles River. Copulation was determined by the presence of a vaginal plug the morning after mating, and embryonic day 0.5 (e0.5) was defined as noon of that day. TGCs and embryos were dissected in 1× PBS (1∶10 10× PBS, pH = 7.4; Gibco) and stored on ice until further processing. After removal of the decidua, parietal TGCs of the mural trophectoderm [15] were dissected away from the placental disk, and, when possible, Reichert's membrane (Figure S1A). TGCs were identified by their extremely large cell size (Figure 1A). Using single-nucleotide polymorphism data from F1 crosses, TGCs were predicted to have, at the most, approximately 5% contamination by maternal cells (Hannibal & Baker, unpublished data). Placental disk tissue was gathered from e13.5 placental disks after the removal of the decidua and obvious parietal TGCs. For gathering 2N genomic DNA, at e8.0, the entire embryo was collected; at e9.5, the embryo body, after removal of obvious organs and head (removed at otic vesicle), was collected; and at later stages, limbs, or a mixture of limbs and the tail, were collected (Figure S1A).

### Nuclear staining

For confocal imaging, TGCs/embryos were fixed in 4% paraformaldehyde at 4°C overnight. Samples were stained with 0.5 µg/mL DAPI (Life Technologies) in 1× PBS overnight, washed in 50% glycerol/1× PBS and stored in 70% glycerol/1× PBS. Confocal images were taken on a Leica DM IRE2 inverted microscope using the Leica SP2 software package, located in the Stanford Cell Sciences Imaging Facility.

### Cell culture

Trophoblast stem cells were cultured as described in Chuong et al. [31] following [27]. TS cells were differentiated into parietal TGCs by replacing the FGF, Activin and Heparin in the media with retinoic acid [27], [28]. Mature TGCs are seen after 4–6 days of differentiation [26] and were collected on days 3, 5 and 7. TGCs/TS cells were further isolated for aCGH by placing cultured cells over a two-step density gradient (1.5% BSA over 3% BSA in a 15 mL tube; Figure S1B). TGCs sank to the bottom of the tube while the smaller TS cells stayed in the upper fraction.

The embryonic stem cell line CGR8 is a germ-line competent cell line established from the inner cell mass of a 129 e3.5 male pre-implantation embryo [45]. ES cells were cultured feeder-free on 0.1% gelatin coated plates. The ES cell medium was prepared by supplementing knockout DMEM (Invitrogen) with 15% FBS, 1 mM glutamax, 0.1 mM nonessential amino acids, 1 mM sodium pyruvate, 0.1 mM 2-mercaptoethanol, penicillin/streptomycin, and 1000 units of leukemia inhibitory factor (LIF; Millipore). Cell culture was maintained at 37°C with 5% CO2.

Megakaryocytes were derived and cultured as described in [46]. Briefly, fetal livers were dissected from e13.5 C57BL/6 embryos in Hanks' Balanced Salt Solution and placed in DMEM with 10% FBS supplemented with 100 ug/mL penicillin-streptomycin (Invitrogen). Livers were pooled based on sex of the embryo (males pooled and females pooled separately). To make a single cell solution, livers were aspirated through a progression of 18G, 21G and 23G needles. To promote differentiation into megakaryocytes, cells were cultured for five days in media containing thrombopoietin (TPO; R&D Systems) at 37°C with 5% CO2. Successful differentiation was identified by 1) the presence of large cells (megakaryocytes) and by 2) FACS to confirm up to 32N ploidy. For FACS, propidium iodide stained samples were run on a Cytek DxP10 modified Facscan (Cytek Technologies, BD Biosciences) using the blue laser. Approximately 10,000 events per sample were collected. Data was analyzed using FlowJo (Treestar, Inc.). Megakaryocytes were isolated for aCGH by placing cultured cells over a two-step density gradient (1.5% BSA over 3% BSA in a 15 mL tube; Figure S1B). Megakaryocytes sank to the bottom of the tube while smaller, undifferentiated, cells stayed in the upper fraction.

### ArrayCGH and whole genome sequencing

Genomic DNA was extracted from fresh tissue and cultured cells using the DNeasy Blood & Tissue Kit (Qiagen). Before column purification, *in vivo* and *in vitro* samples were digested with proteinase-K (600 mAU/ml solution or 40 mAU/mg protein) overnight and for 10 minutes, respectively, at 56°C, followed by a 4 minute incubation with RNase A (100 mg/mL; Qiagen DNeasy Blood & Tissue Kit). If necessary, DNA was further concentrated via ethanol/sodium acetate precipitation following standard protocols.

For arrays performed on DNA from TGCs, placental disks and embryonic controls, genomic DNA from two individuals in the same litter were pooled for each condition. For megakaryocyte arrays, cells derived from 5–6 livers from a single litter were pooled for each condition. For controls for the megakaryocyte array, three embryos (subset of the litter from which livers were collected from) were pooled for each condition. For arrays performed on DNA from cultured cells, two replicates from different passages were used (5 million cells each). For each condition, approximately 4 µg DNA was sent to the Biomedical Genomics Core at the Research Institute at Nationwide Children's Hospital (Columbus, OH) for processing with the SurePrint G3 Mouse CGH Microarray Kit, 4×180 k (Agilent). For all arrays performed on DNA from *in vivo* tissue, to ensure that the arrays detect copy number variation, duplicates consist of 1) female test versus male control and 2) male test versus female control.

aCGH data was analyzed using the R/Bioconductor package cghFLasso, which utilizes reference arrays in conjunction with a FDR [21]. An FDR of 0.0001 was used in order to examine all of the autosomes simultaneously. To determine which array to use as the reference, several analyses were performed. The TS versus ES array exhibited specific CNVs, presumably due to genomic adaptations to culturing [22]. The megakaryocytes displayed only a small region of overrepresentation and the placental disk array did not display any CNVs (FDR = 0.0001). However, as the placental disk has a small amount of underrepresentation in reproducible areas of the genome (FDR≥0.05), the megakaryocyte array was used as the reference for the remainder of the analyses. aCGH data was plotted using cghFLasso [21]. For comparison with data from Sher et al. [19], data was retrieved from Gene Expression Omnibus series: GSE45787. To compare aCGH data from Sher et al. to data presented here, results were plotted using a custom R/Bioconductor program.

For WGS, for stages e9.5 and older, genomic DNA from one individual was used for each replicate, and for stage e8.0, 5–7 individuals from one litter were used for each replicate. Libraries for WGS were prepared from 40–50 ng genomic DNA using the Nextera TruSeq Dual Index Paired End Kit (Illumina) following manufacturer's instructions with the following modification: the Qiagen MinElute Reaction Cleanup Kit (Qiagen) was used to cleanup Tagmented DNA. Library quality was assessed using Qubit and Bioanalyzer, and sequenced on the Illumina HiSeq 2000 at approximately 10× coverage (Table S4) at the Stanford Center for Genomics and Personalized Medicine. 101 bps from each of the paired-ends were sequenced and sequencing reads were aligned using either the DNAnexus mapper [47] or the Novocraft Novoalign program against the mouse reference genome (mm9). Data was analyzed using custom R/Bioconductor programs and SMASH [34]. To compare aCGH versus WGS data, results were plotted using a custom R/Bioconductor program.

The final UR domain list was generated using e9.5 WGS data and a custom R/Bioconductor program with the following criteria: neighboring data points with normalized log 2 ratio of TGCs/embryo ≤−0.3. These criteria were decided upon based on the program CNVnator [24], which, while identifying UR domains with both large and small degrees of underrepresentation at a p-value of 0.01, systematically missed UR domains that are closely spaced together, which our program rectifies.

### Enrichment statistics

To calculate the significance of overlap between datasets, a binomial test was used to determine whether the observed overlap for the datasets was significantly greater than an expected overlap based on the average of 1,000 randomized datasets [31]. To randomize each dataset, regions were shuffled within bins according to their chromosomal distribution and distance from gene transcriptional start sites (including 1 kb, 10 kb, 100 kb, 1,000 kb, and >1,000 kb bins).

### 3SEQ

Total RNA was extracted from fresh *in vivo* tissue by homogenizing in TRIzol Reagent (Life Technologies/Ambion) and total RNA was prepared following manufacturer's instructions. Total RNA from three individuals from the same litter were combined to make each library. mRNA was isolated from 10–20 µg of total RNA using Dynabeads Oligo(dT)_25_ (Life Technologies/Ambion). 3SEQ Libraries were prepared from mRNA following [30]. Briefly, mRNA was heat sheared for 7.5 minutes to produce an average fragment size range of 100–400 bp, then used to generate cDNA libraries using a custom oligo dT primer containing Illumina-compatible adapter sequence. cDNA fragments were end-repaired and ligated to standard Illumina adapters. Size-selection was performed using E-gel SizeSelect agarose gels (Invitrogen), products were PCR amplified for 15 cycles and purified using Ampure XP beads (Beckman Coulter). Library quality was assessed using Qubit and Bioanalyzer, and sequenced on the Genome Analyzer IIx at the Stanford Center for Genomics and Personalized Medicine.

Total RNA was extracted and 3SEQ libraries were constructed for cultured TGCs as described in Chuong et al. [31]. Two replicates from different passages (10 million cells each) were used. 3SEQ data for TS cells was retrieved from Gene Expression Omnibus series: GSE42207 [31].

Sequences were aligned to the mouse (mm9) genome using the DNAnexus mapper [47] and raw counts for sense reads were analyzed using Unipeak 1.0 [48]. Regions of transcription were associated with the nearest ENSEMBL gene 3′ UTR within 5 kb. Data were normalized and expression levels were analyzed using the R/Bioconductor package DESeq [49].

### ChIP-seq

ChIP-seq and ChIP-seq analysis were performed as described in Chuong et al. [31] using the ChIP Assay kit (Millipore) following manufacturer's instructions. Briefly, 20 million cultured TGCs were cross-linked in 2% formaldehyde for 15 minutes, and sonicated for 12 cycles (30 seconds on/off) at 60% amplitude to produce a fragment range of 300–600 bp. Immunoprecipitation was performed with 2–5 µg of antibody (H3K4me3: ActiveMotif, 39159; H3K27me3: ActiveMotif, 39535; H3K27ac: Abcam, ab4729; H3K9me3: Abcam, ab8898; H3K4me1: Abcam, ab8895) conjugated to 50 µl of protein G Dynabeads (Invitrogen) overnight. Following washing and elution of DNA per manufacturer's instructions, libraries were prepared using the Illumina genomic DNA preparation kit using barcoded linker adapters, and sequenced on the Illumina HiSeq 2000 at the Stanford Center for Genomics and Personalized Medicine. ChIP-Seq data for TS cells was retrieved from Gene Expression Omnibus series: GSE42207 [31].

High-quality reads were aligned to the mm9 genome assembly using BWA 0.5.9 [50], retaining only unique alignments. Peaks were called using MACS2 2.0.10 [32]. The “bigwig_correlation” script from the Cistrome package [51] was used to generate genome-wide correlation plots between ChIP profiles and underrepresented profiles.

### Replication timing

Cultured TS cells were incubated for two hours at 37°C in the dark with a final concentration of 100 µM BrdU (Sigma Aldrich B5002). Genome-wide replication timing was analyzed as previously described [36]. Briefly, cells were dissociated into a single-cell suspension and nuclei were isolated. DNA was subsequently stained with propidium iodide and cells were FACS sorted into early and late S-phase fractions based on their DNA content. DNA from early and late S-phase fractions was purified by immunoprecipitation of the BrdU-substituted nascent DNA (BrdU-IP). Three replicates from different passages (two million cells each) were used. Data was normalized following [36]. The R/bioconductor package DNAcopy was used to define replication timing domains based on the similarity in values (a constant value across a segment defines a domain) [36]. Regions called by DNAcopy were confirmed on the genome browser. The “bigwig_correlation” script from the Cistrome package [51] was used to generate genome-wide correlation plots between replication timing profiles and underrepresented profiles.

### Accession codes and data availability

SuperSeries Gene Expression Omnibus (GEO) accession number for aCGH, 3SEQ, ChIP-Seq, and replication timing data: GSE50585.

Smoothed replication timing data can also be found at: http://www.replicationdomain.com/

BioProject accession number for WGS: PRJNA213010

## Supporting Information

Figure S1 Collection of polyploid and 2N cells. **A**. Collection of TGCs and 2N embryonic tissue *in vivo*. After removal of the decidua, parietal TGCs of the mural trophectoderm were dissected away from the placental disk. While parietal TGCs surround the conceptus at earlier stages, at later stages they are only present around the placental disk, at the edge of the placental disk, and as a “belt” around the embryo. At later stages, only the TGCs around the edge of the placental disk were collected. For gathering 2N genomic DNA, at e8.0, the entire embryo was collected; at e9.5, the embryo body, after removal of obvious organs and head (removed at otic vesicle), was collected; and at later stages, limbs, or a mixture of limbs and the tail, were collected. Left: cross-section of conceptus with maternal decidua; middle: conceptus without maternal decidua, “X” marks discarded tissue; right: remaining TGCs and embryonic tissue used for experiments. Dashed box in e13.5: region of placental disk used for placental disk aCGH. Yellow: parietal TGCs; gray: other embryonic/extraembryonic tissue; pink: maternal decidua. **B**. Collection of polyploid and 2N cells *in vitro*. After culturing under conditions for either 2N cells or polyploid cells, the desired cells were further isolated by placing them over a two-step density gradient (1.5% BSA over 3% BSA). Polyploid cells sank to the bottom, while the smaller 2N cells stayed in the upper fraction.
(TIF)

Figure S2 e9.5 TGC, placental disk and megakaryocyte aCGH. Plots comparing position along all autosomes to the NLog2 Ratio of array intensity of test vs. control. Red: e9.5 TGC vs. embryo; purple: placental disk vs. embryo; blue: megakaryocyte vs. embryo. Two biological replicates are plotted for each cell type. Dashed line: FDR = 0.0001.
(TIF)

Figure S3 Comparison of e9.5 TGC aCGH with Sher et al. Plots comparing position along all autosomes to the NLog2 Ratio of array intensity of test vs. control. Red: e9.5 TGC vs. embryo (this study); purple: placental disk vs. embryo (this study); teal: e9.5 TGC vs. embryo (Sher et al. [19]). Two biological replicates are plotted for each cell type. Dashed line: FDR = 0.0001.
(TIF)

Figure S4 Comparison of e9.5 aCGH and WGS. Plots comparing position along all autosomes to the NLog2 Ratio of array intensity (aCGH) and sequence coverage (WGS) of TGCs vs. embryos. Red: e9.5 WGS; blue: e9.5 aCGH. Two biological replicates are plotted for each platform (LitterA shown for WGS).
(TIF)

Figure S5 e9.5 WGS. Plots comparing position along all autosomes to the NLog2 Ratio of sequence coverage of TGCs vs. embryos for six individuals. In general, outside of the UR domains, LitterA-01 does not trend as closely with the others. This is mainly due to variability in the embryo, as TGCs from LitterA-01 trends more closely with the others when compared to its litter-mate embryo from LitterA-02, although see chromosome 5 (asterisk) for a striking exception. Orange: LitterA-01; steel blue: LitterA-02; red: LitterB-01; sky blue: LitterB-03; magenta: LitterC-02; green: LitterC-07. Dashed orange line: TGCs from LitterA-01 compared to the embryo from LitterA-02. Dashed black line: cut-off for significance.
(TIF)

Figure S6 Comparison of e8.0 and e9.5 WGS. Plots comparing position along all autosomes to the NLog2 Ratio of sequence coverage of TGCs vs. embryos. Red: e9.5; blue: e8.0. Two biological replicates are plotted for each stage (LitterA shown for WGS). Dashed line: cut-off for significance.
(TIF)

Figure S7 Comparison of e9.5–e16.5 aCGH. Plots comparing position along all autosomes to the NLog2 Ratio of array intensity of TGC vs. embryo. Red: e9.5; blue: e11.5; green: e13.5; orange: e16.5. Two biological replicates are plotted for each stage. Dashed line: FDR = 0.0001.
(TIF)

Figure S8 aCGH for *in vitro* TGCs differentiated 3, 5 and 7 days. Plots comparing position along all autosomes to the NLog2 Ratio of array intensity of TGC vs. embryo (e9.5) and TGC vs. TS cells (day 3, 5, and 7). Red: e9.5 (*in vivo*); blue: day 3 (*in vitro*); green: day 5 (*in vitro*); orange: day 7 (*in vitro*). Two biological replicates are plotted for each cell type. Dashed line: FDR = 0.0001.
(TIF)

Figure S9 Models of UR domain formation. **A**. Deletion detection using paired-end reads. **Top:** A sequencing library is made from a genome containing a deletion between A and B. Some of these reads will span the deleted region (red arrowheads). Paired-end reads (red arrowheads) are 101 bp reads flanking an approximately 500 bp unsequenced region (red line). **Bottom:** Sequenced reads (red arrowheads) are aligned to the reference genome, which does not contain the deletion between A and B. If the distance between the paired-end reads is greater than the expected insert size (“discordant” paired-end read), then this indicates a deletion in the sequenced genome compared to the reference genome. Here, instead of mapping 500 bps apart, the paired-ends map 10,000 bps apart (red dotted line), suggesting a deletion. **B**. Models of UR domain formation. UR domains are in red. A, B, C mark regularly represented regions flanking UR domains. **Top:** Trace of NLog2 ratio of WGS data. WGS data suggests UR domains are underrepresented by approximately 50%. **Model 1:** UR domains are deleted from the genome by 50%. UR domains are present in half the chromosomes, but deleted from the other half. **Model 2:** UR domains are underreplicated by 50%. UR domains are underreplicated regions flanked by slowed or stalled replication forks. In this scenario, UR domains are continuous with regularly represented regions, therefore, UR domains would not be deleted from the genome and deletions would not be detected.
(TIF)

Table S1 UR domains in e9.5 TGCs (aCGH). UR domain location and size in e9.5 TGCs based on aCGH data. UR domains were called using the program cghFLasso [21] with a FDR of 0.0001. Asterisks mark UR domains found in both aCGH biological replicates, but only four to five out of the six WGS biological replicates.
(XLSX)

Table S2 Underrepresentation/Overrepresentation in e9.5 TGCs, TS cells and Megakaryocytes (Placenta Disk Array as Control). Summary of array results for the following conditions: TGCs vs. embryos, ES cells vs. TS cells, and megakaryocytes vs. embryos. The cghFLasso program utilizes reference arrays to call underrepresentations/overrepresentations [21]. Therefore, this table summarizes calls when the TGCs/embryo, ES/TS cells, and megakaryocyte/embryo arrays were compared to the placenta disk/embryo arrays. A underrepresentation or overrepresentation was only called if present in both biological replicates at an FDR = 0.0001. Del/Dupl = underrepresentation or overrepresentation, in TGCs, TS cells or magakaryocytes. There are only underrepresented regions (UR domains) called for e9.5 TGCs. There is only one overrepresented region in megakaryocytes (containing the following annotated genes: Pisd-ps1, Sfi1). This region is located at the end of a chromosome (Chr 11), which suggests that it is an artifact. As both cultured TS and ES cells may have underrepresentations/overrepresentations due to culturing [22], underrepresentations/overrepresentations in TS cells could also be overrepresentations/underrepresentations in ES cells. Putative underreplicated regions in TS cells generally do not correspond to UR domains in e9.5 TGCs.
(XLSX)

Table S3 Underrepresentation/Overrepresentation in e9.5 TGCs, TS cells and the Placenta Disk (Megakaryocyte Array as Control). Summary of array results for the following conditions: TGCs vs. embryo, ES cells vs. TS cells, and placenta disk cells vs. embryo. The cghFLasso program utilizes reference arrays to call underrepresentations/overrepresentations [21]. Therefore, this table summarizes calls when the TGCs/embryo, ES/TS cells, and placenta disk/embryo arrays were compared to the megakaryocyte/embryo arrays. A underrepresentation or overrepresentation was only called if present in both biological replicates at an FDR = 0.0001. Del/Dupl = underrepresentation or overrepresentation in TGCs, TS cells or placenta disk. There are only underrepresented regions (UR domains) called for e9.5 TGCs. There are no underrepresentations or overrepresentations called in the placenta disk. As both cultured TS and ES cells may have underrepresentations/overrepresentations due to culturing [22], underrepresentations/overrepresentations in TS cells could also be overrepresentations/underrepresentations in ES cells. Putative underreplicated regions in TS cells generally do not correspond to UR domains in e9.5 TGCs.
(XLSX)

Table S4 Whole genome sequencing statistics. Number of mapped reads and coverage for each sequenced sample. Coverage was calculated by the following formula: [Read length (101 bps) * Read number (mapped reads for a specific sample)]/Size of mouse haploid genome (2.7×10^9^ bps).
(XLSX)

Table S5 UR domains in six e9.5 individuals (WGS). UR domain locations in six e9.5 individual from three different litters (A, B and C). UR domains common to all six samples are in bold. Samples with the least number of UR domains have a subset of the UR domains found in the samples with the most UR domains. In addition, the size of the shared UR domains are smaller in the samples with fewer UR domains.
(XLSX)

Table S6 Late-replicating regions containing UR domains. Late-replicating regions that contain UR domains. Replication timing regions defined by the R/Bioconductor program DNAcopy [36]. Asterisk marks the one UR domain that does not fall completely within the late-replicating region defined by DNA copy, but that is entirely late-replicating when viewed on the UCSC genome browser.
(XLSX)
