# Supplementary figures and images for: Copy Number Variation Is a Fundamental Aspect of the Placental Genome
Source: PLoS Genet. 2014 May 1;10(5):e1004290. doi: 10.1371/journal.pgen.1004290 (PMC4006706; doi:10.1371/journal.pgen.1004290)

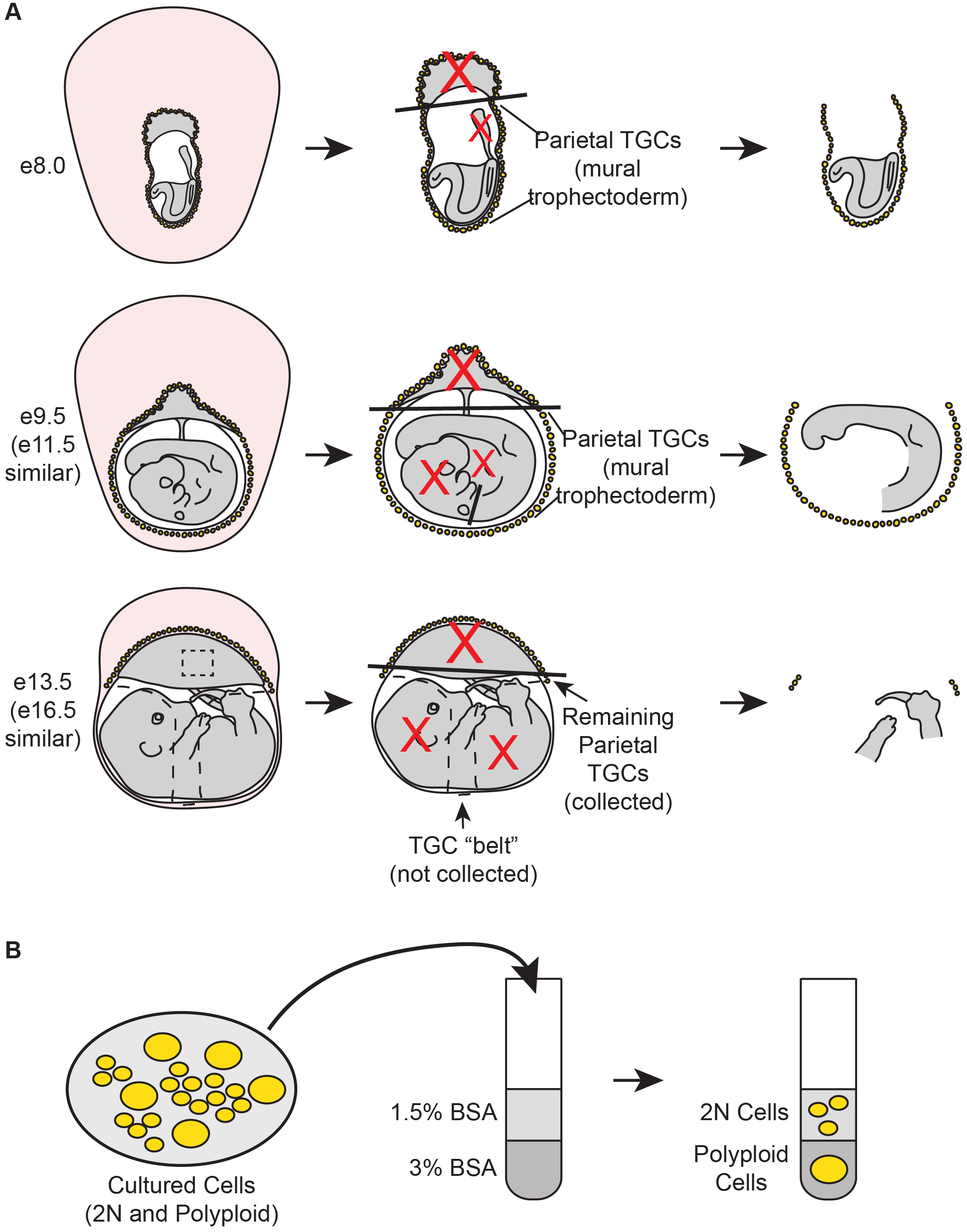

Supplement: Figure S1 — Collection of polyploid and 2N cells. A. Collection of TGCs and 2N embryonic tissue in vivo. After removal of the decidua, parietal TGCs of the mural trophectoderm were dissected away from the placental disk. While parietal TGCs surround the conceptus at earlier stages, at later stages they are only present around the placental disk, at the edge of the placental disk, and as a “belt” around the embryo. At later stages, only the TGCs around the edge of the placental disk were collected. For gathering 2N genomic DNA, at e8.0, the entire embryo was collected; at e9.5, the embryo body, after removal of obvious organs and head (removed at otic vesicle), was collected; and at later stages, limbs, or a mixture of limbs and the tail, were collected. Left: cross-section of conceptus with maternal decidua; middle: conceptus without maternal decidua, “X” marks discarded tissue; right: remaining TGCs and embryonic tissue used for experiments. Dashed box in e13.5: region of placental disk used for placental disk aCGH. Yellow: parietal TGCs; gray: other embryonic/extraembryonic tissue; pink: maternal decidua. B. Collection of polyploid and 2N cells in vitro. After culturing under conditions for either 2N cells or polyploid cells, the desired cells were further isolated by placing them over a two-step density gradient (1.5% BSA over 3% BSA). Polyploid cells sank to the bottom, while the smaller 2N cells stayed in the upper fraction. (TIF) [file pgen.1004290.s001.tif]

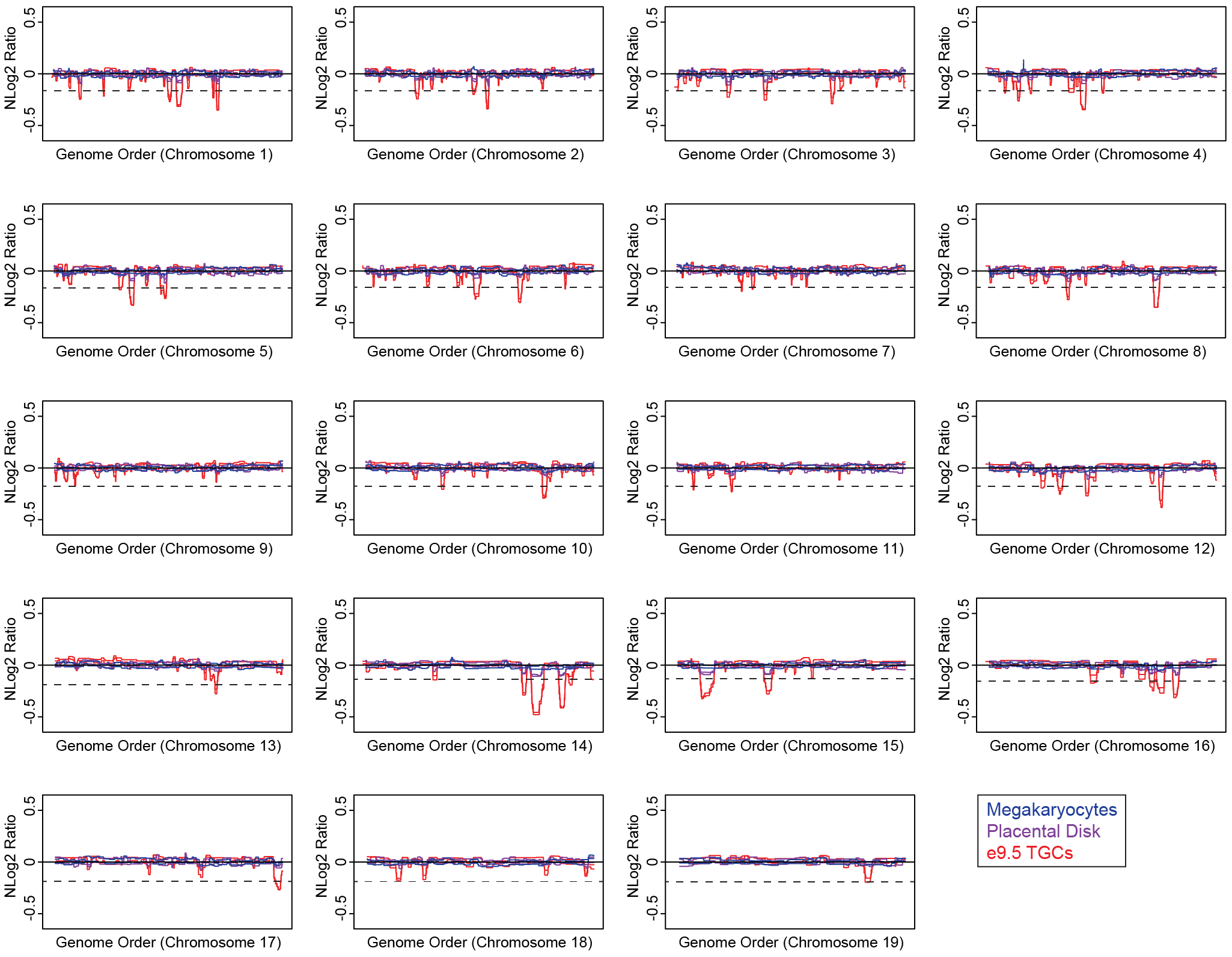

Supplement: Figure S2 — e9.5 TGC, placental disk and megakaryocyte aCGH. Plots comparing position along all autosomes to the NLog2 Ratio of array intensity of test vs. control. Red: e9.5 TGC vs. embryo; purple: placental disk vs. embryo; blue: megakaryocyte vs. embryo. Two biological replicates are plotted for each cell type. Dashed line: FDR = 0.0001. (TIF) [file pgen.1004290.s002.tif]

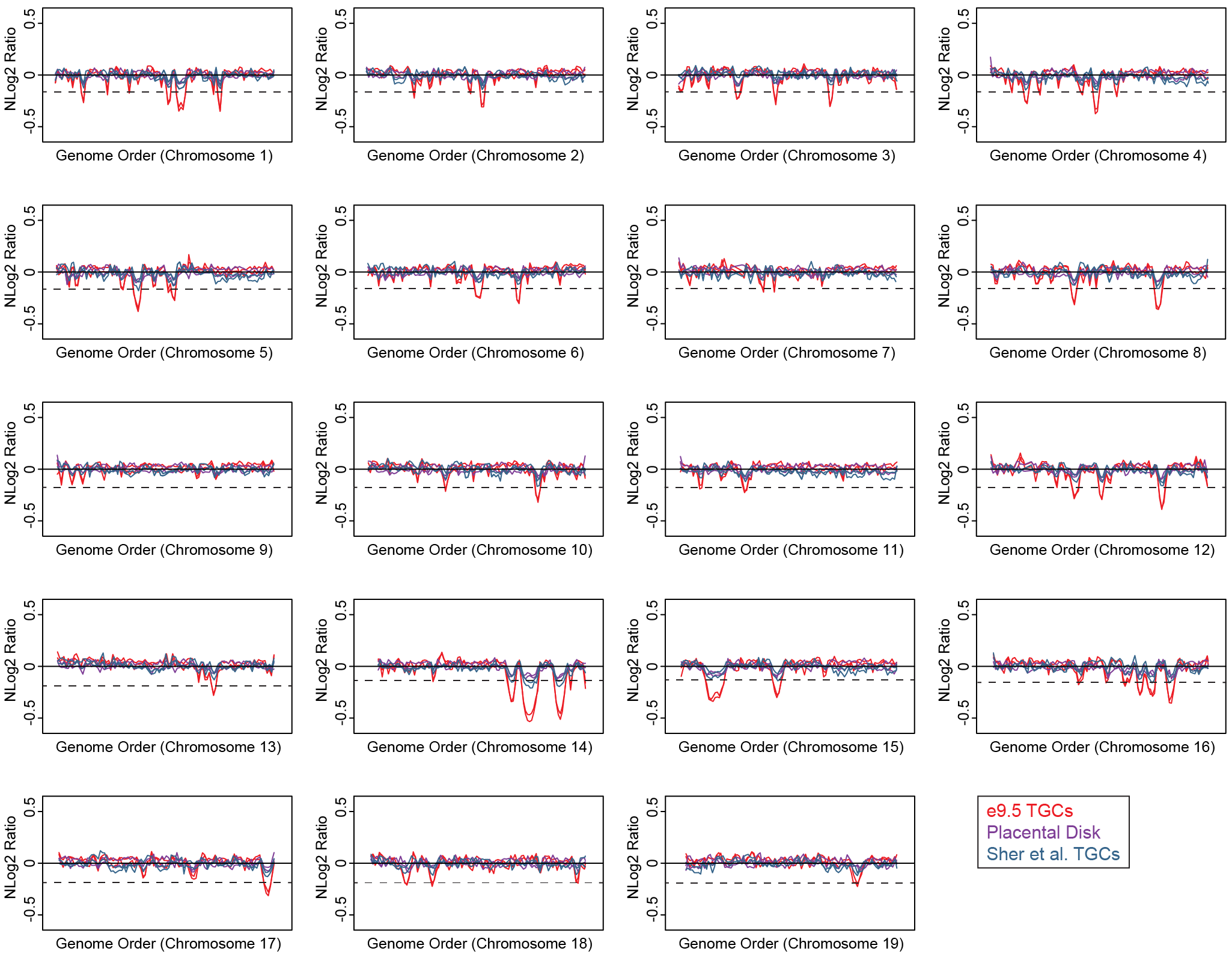

Supplement: Figure S3 — Comparison of e9.5 TGC aCGH with Sher et al. Plots comparing position along all autosomes to the NLog2 Ratio of array intensity of test vs. control. Red: e9.5 TGC vs. embryo (this study); purple: placental disk vs. embryo (this study); teal: e9.5 TGC vs. embryo (Sher et al. [19]). Two biological replicates are plotted for each cell type. Dashed line: FDR = 0.0001. (TIF) [file pgen.1004290.s003.tif]

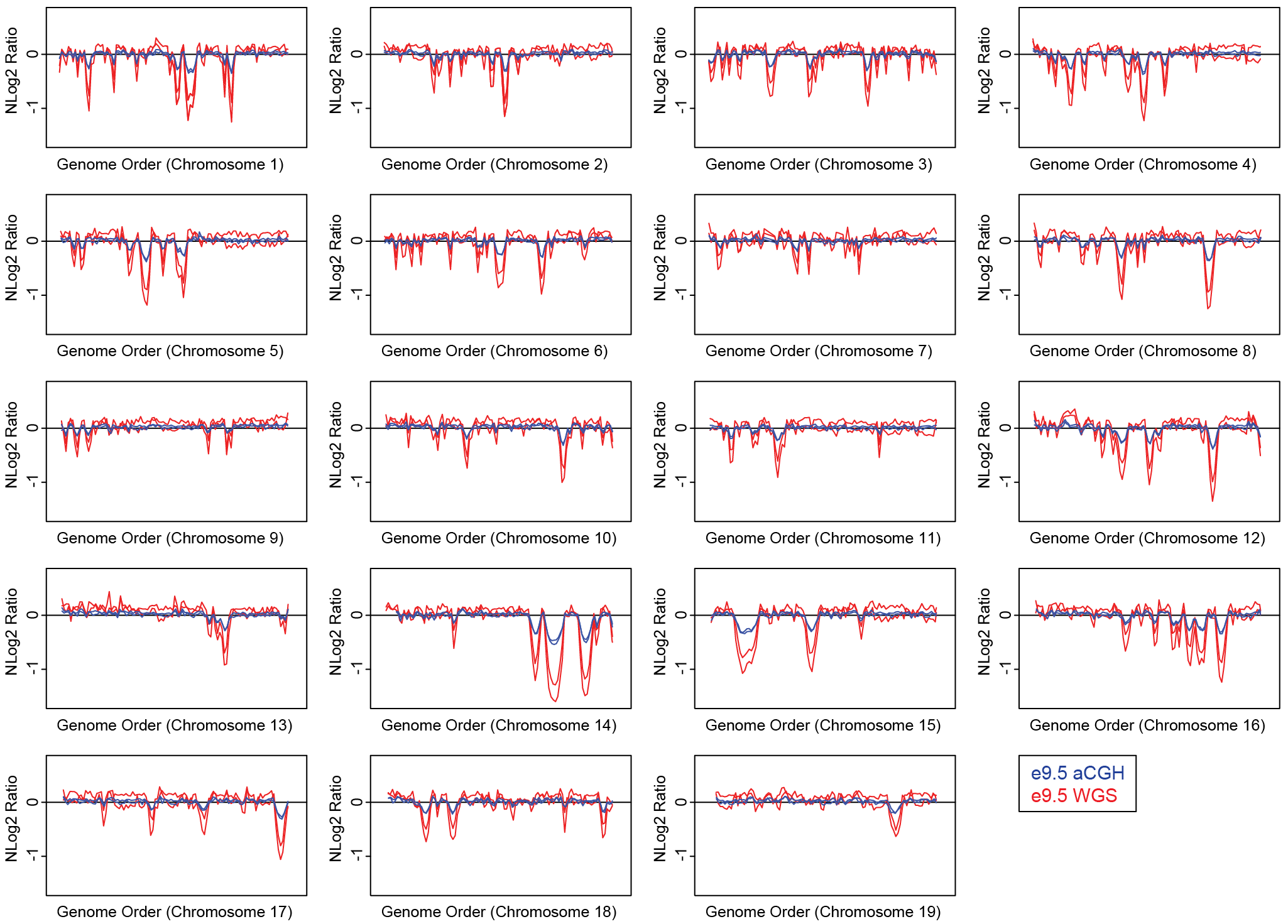

Supplement: Figure S4 — Comparison of e9.5 aCGH and WGS. Plots comparing position along all autosomes to the NLog2 Ratio of array intensity (aCGH) and sequence coverage (WGS) of TGCs vs. embryos. Red: e9.5 WGS; blue: e9.5 aCGH. Two biological replicates are plotted for each platform (LitterA shown for WGS). (TIF) [file pgen.1004290.s004.tif]

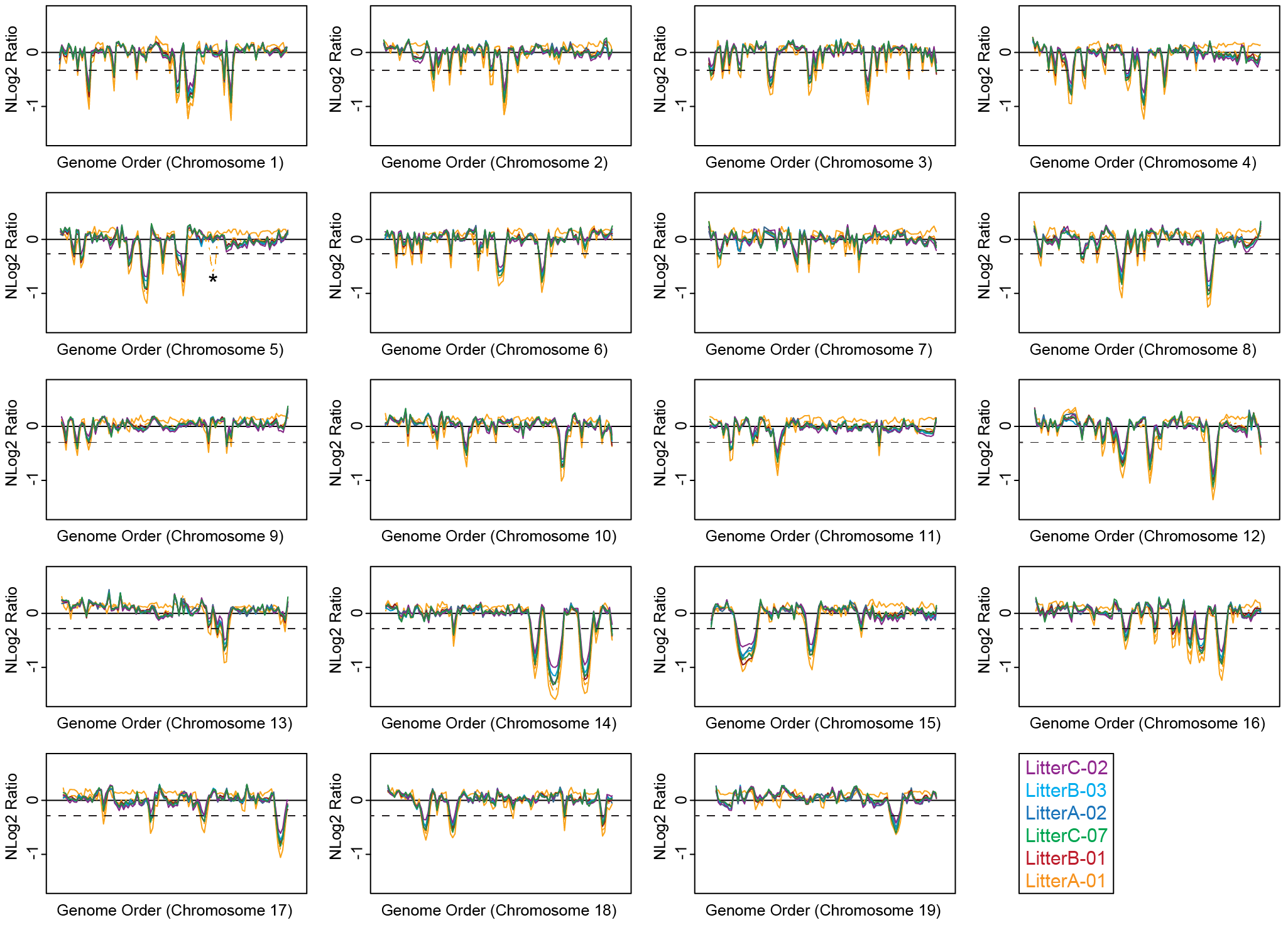

Supplement: Figure S5 — e9.5 WGS. Plots comparing position along all autosomes to the NLog2 Ratio of sequence coverage of TGCs vs. embryos for six individuals. In general, outside of the UR domains, LitterA-01 does not trend as closely with the others. This is mainly due to variability in the embryo, as TGCs from LitterA-01 trends more closely with the others when compared to its litter-mate embryo from LitterA-02, although see chromosome 5 (asterisk) for a striking exception. Orange: LitterA-01; steel blue: LitterA-02; red: LitterB-01; sky blue: LitterB-03; magenta: LitterC-02; green: LitterC-07. Dashed orange line: TGCs from LitterA-01 compared to the embryo from LitterA-02. Dashed black line: cut-off for significance. (TIF) [file pgen.1004290.s005.tif]

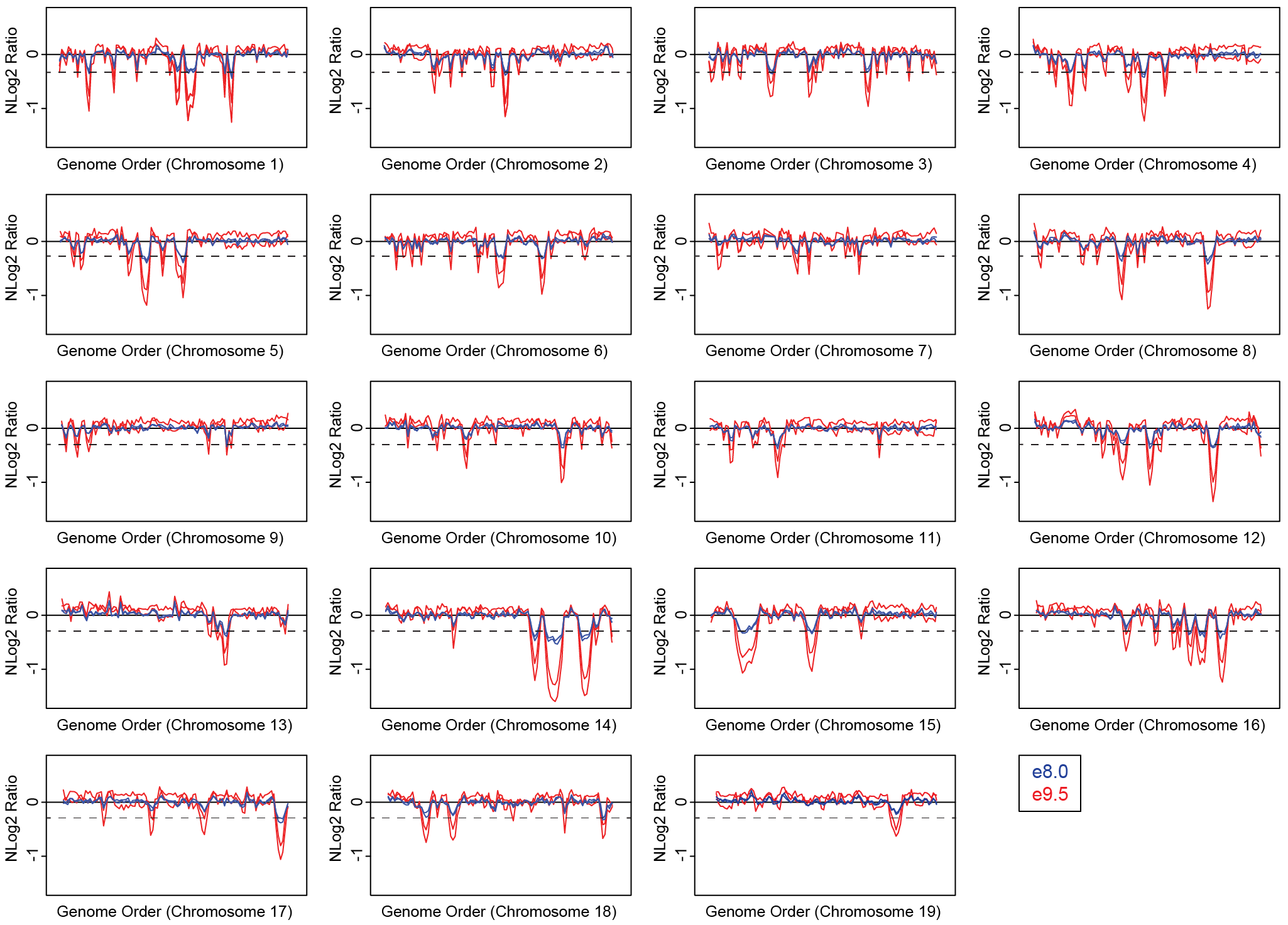

Supplement: Figure S6 — Comparison of e8.0 and e9.5 WGS. Plots comparing position along all autosomes to the NLog2 Ratio of sequence coverage of TGCs vs. embryos. Red: e9.5; blue: e8.0. Two biological replicates are plotted for each stage (LitterA shown for WGS). Dashed line: cut-off for significance. (TIF) [file pgen.1004290.s006.tif]

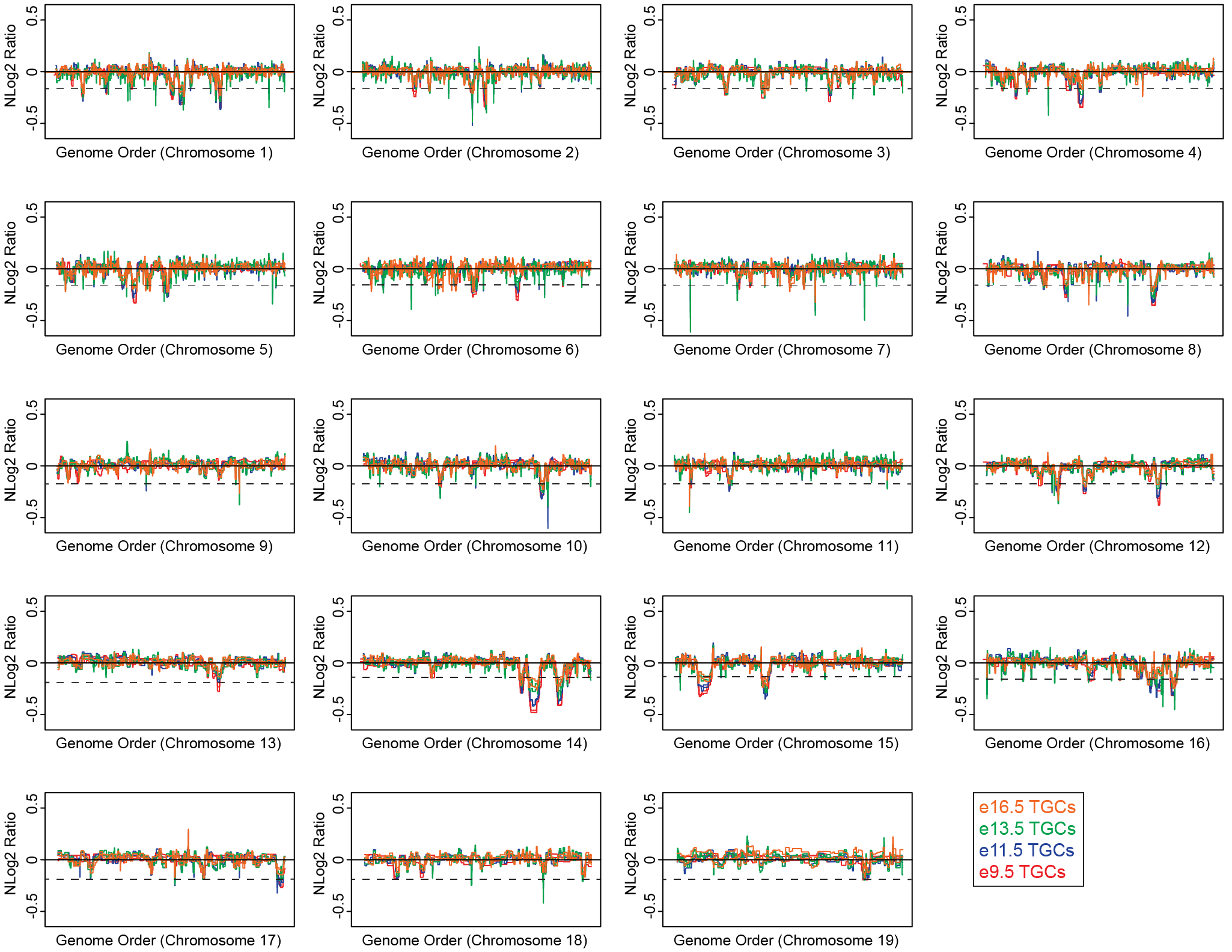

Supplement: Figure S7 — Comparison of e9.5–e16.5 aCGH. Plots comparing position along all autosomes to the NLog2 Ratio of array intensity of TGC vs. embryo. Red: e9.5; blue: e11.5; green: e13.5; orange: e16.5. Two biological replicates are plotted for each stage. Dashed line: FDR = 0.0001. (TIF) [file pgen.1004290.s007.tif]

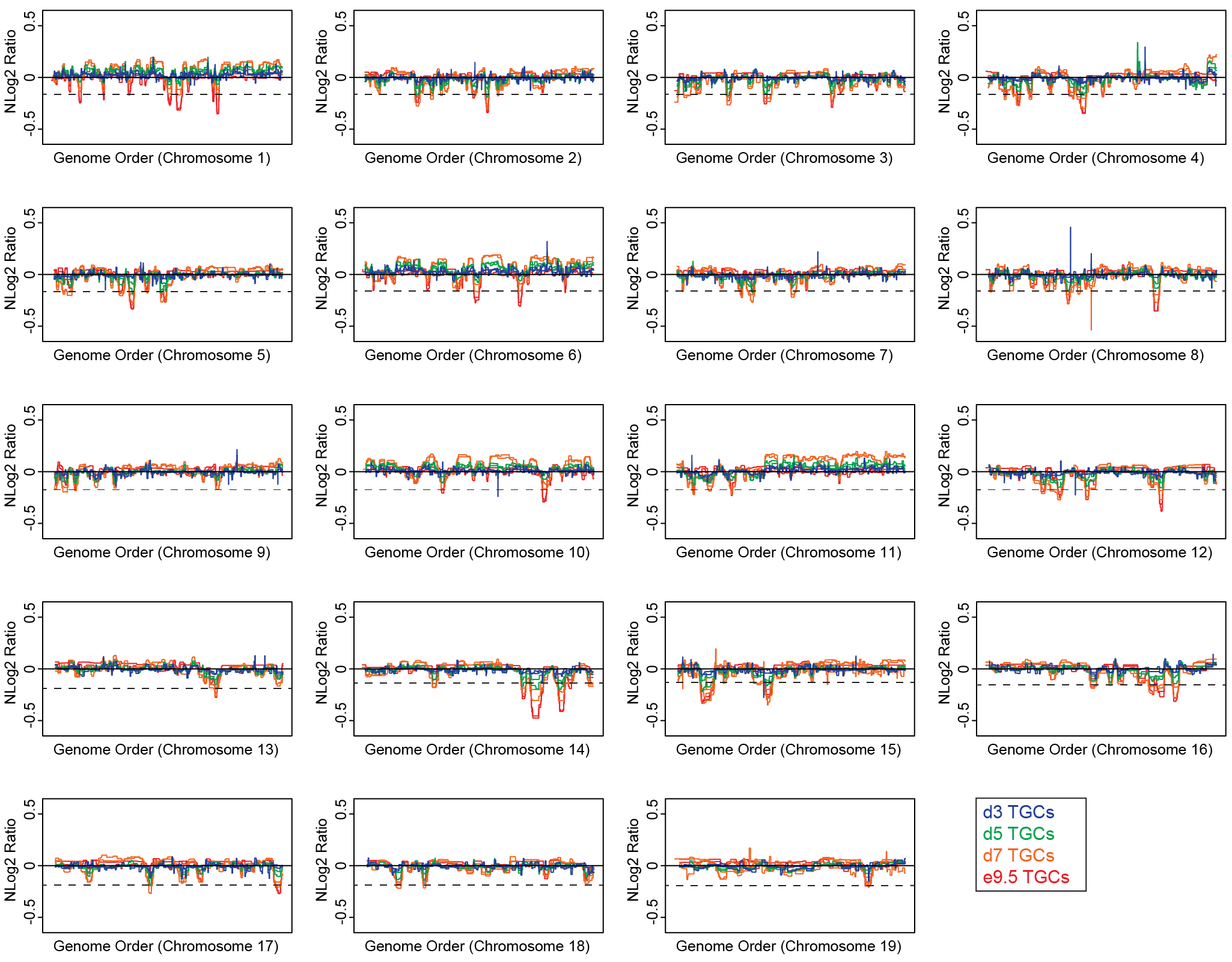

Supplement: Figure S8 — aCGH for in vitro TGCs differentiated 3, 5 and 7 days. Plots comparing position along all autosomes to the NLog2 Ratio of array intensity of TGC vs. embryo (e9.5) and TGC vs. TS cells (day 3, 5, and 7). Red: e9.5 (in vivo); blue: day 3 (in vitro); green: day 5 (in vitro); orange: day 7 (in vitro). Two biological replicates are plotted for each cell type. Dashed line: FDR = 0.0001. (TIF) [file pgen.1004290.s008.tif]

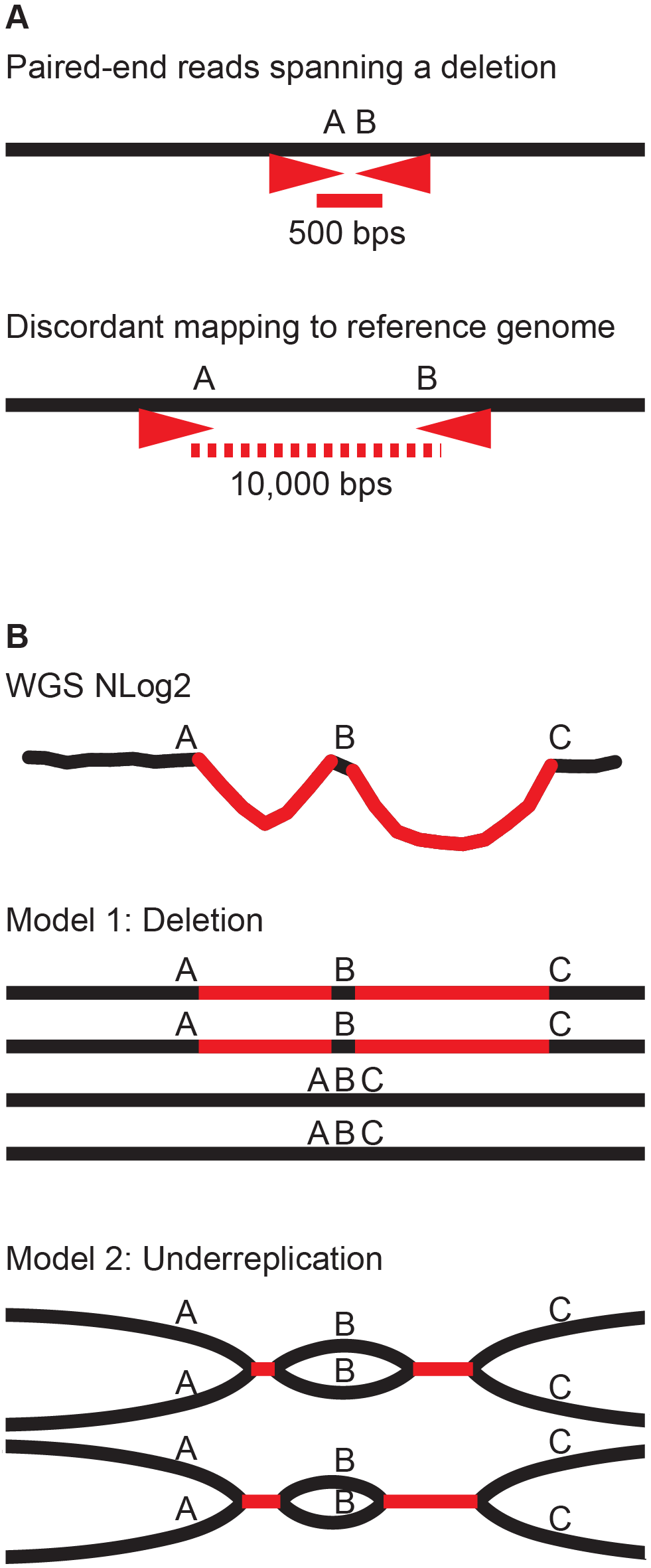

Supplement: Figure S9 — Models of UR domain formation. A. Deletion detection using paired-end reads. Top: A sequencing library is made from a genome containing a deletion between A and B. Some of these reads will span the deleted region (red arrowheads). Paired-end reads (red arrowheads) are 101 bp reads flanking an approximately 500 bp unsequenced region (red line). Bottom: Sequenced reads (red arrowheads) are aligned to the reference genome, which does not contain the deletion between A and B. If the distance between the paired-end reads is greater than the expected insert size (“discordant” paired-end read), then this indicates a deletion in the sequenced genome compared to the reference genome. Here, instead of mapping 500 bps apart, the paired-ends map 10,000 bps apart (red dotted line), suggesting a deletion. B. Models of UR domain formation. UR domains are in red. A, B, C mark regularly represented regions flanking UR domains. Top: Trace of NLog2 ratio of WGS data. WGS data suggests UR domains are underrepresented by approximately 50%. Model 1: UR domains are deleted from the genome by 50%. UR domains are present in half the chromosomes, but deleted from the other half. Model 2: UR domains are underreplicated by 50%. UR domains are underreplicated regions flanked by slowed or stalled replication forks. In this scenario, UR domains are continuous with regularly represented regions, therefore, UR domains would not be deleted from the genome and deletions would not be detected. (TIF) [file pgen.1004290.s009.tif]
